# Identification and characteristics of *SnRK* genes and cold stress-induced expression profiles in *Liriodendron chinense*

**DOI:** 10.1186/s12864-022-08902-0

**Published:** 2022-10-18

**Authors:** Rongxue Li, Yasmina Radani, Baseer Ahmad, Ali Movahedi, Liming Yang

**Affiliations:** 1 College of Biology and the Environment, Nanjing Forestry University, Nanjing, 210037, China; 2 Muhammad Nawaz Sharif University of Agriculture, Multan, Punjab 25000 Pakistan

**Keywords:** Cold stress, Expression pattern, Genome-wide identification, *L.chinense*, *SnRK*

## Abstract

**Background:**

The sucrose non-fermenting 1 (SNF1)-related protein kinases (SnRKs) play a vivid role in regulating plant metabolism and stress response, providing a pathway for regulation between metabolism and stress signals. Conducting identification and stress response studies on *SnRKs* in plants contributes to the development of strategies for tree species that are more tolerant to stress conditions.

**Results:**

In the present study, a total of 30 *LcSnRKs* were identified in *Liriodendron chinense* (*L. chinense*) genome, which was distributed across 15 chromosomes and 4 scaffolds. It could be divided into three subfamilies: *SnRK1*, *SnRK2*, and *SnRK3* based on phylogenetic analysis and domain types. The *LcSnRK* of the three subfamilies shared the same Ser/Thr kinase structure in gene structure and motif composition, while the functional domains, except for the kinase domain, showed significant differences. A total of 13 collinear gene pairs were detected in *L. chinense* and *Arabidopsis thaliana* (*A. thaliana*), and 18 pairs were detected in *L. chinense* and rice, suggesting that the *LcSnRK* family genes may be evolutionarily more closely related to rice. Cis-regulation element analysis showed that *LcSnRKs* were LTR and TC-rich, which could respond to different environmental stresses. Furthermore, the expression patterns of *LcSnRKs* are different at different times under low-temperature stress. *LcSnRK1s* expression tended to be down-regulated under low-temperature stress. The expression of *LcSnRK2s* tended to be up-regulated under low-temperature stress. The expression trend of *LcSnRK3s* under low-temperature stress was mainly up-or down-regulated.

**Conclusion:**

The results of this study will provide valuable information for the functional identification of the *LcSnRK* gene in the future.

**Supplementary Information:**

The online version contains supplementary material available at 10.1186/s12864-022-08902-0.

## Background

Abiotic stress features a genuine effect on plant development and improvement. Plants have evolved complex and precise signal transduction components because of various misfortunes. As major components of the intracellular signal transduction system, protein kinases play a critical part in the stress response. Among them, sucrose non-fermenting 1 (SNF1)-related protein kinase (*SnRK*) is broadly included in different physiological life forms [1–4].

*SnRK* is a serine/threonine protein kinase whose protein has a conserved Ser/Thr protein kinase domain at the N- terminal. Based on phylogenetic analysis of functional domains, the *SnRK* family in plants has been divided into three subfamilies: *SnRK1*, *SnRK2*, and *SnRK3* [5]. The *SnRK1* subfamily includes three domains: kinase domain, UBA domain, and KA1 domain. The UBA domain can mediate the non-covalent interactions of the ubiquitination protein [6]. The KA1 domain can interact with a phosphatase upstream of SnRK1 and other proteins [7–9]. The *SnRK2* and *SnRK3* subfamilies are plant-specific proteins that have been impressively extended in embryonic plants [10]. An extremely conserved kinase domain was contained in the N-terminal of SnRK2s, and an acidic amino acid fragments domain was contained in the C-terminal of SnRK2s [11]. The C-terminal domain comprises two sub-domains, Domain I and Domain II. Domain I is a feature of all members of the *SnRK2* subfamily and must be activated by osmotic stress. Domain II is only specific for ABA-dependent *SnRK2s* and can eventually respond to ABA [12, 13]. *SnRK3* is also known as Calcineurin B-like protein-interacting protein kinases (*CIPK*) as it can bind to CBL to participate in the Ca2+ mediated stress response. Besides a kinase-conservative domain at the N- terminal, CIPK also had a specific NAF domain that could mediate CBL binding and a PPI-conservative domain that could mediate PP2C binding at the C- terminal [14, 15]. It was concluded that *SnRK2* and *SnRK3* were imitated from *SnRK1* [16] and rapidly extended and separated with plant advancement, taking an interest in the interaction of metabolic direction and stress response signaling pathways, thus conferring multiple effects on terrestrial plants corresponding to the stimulation to the external environment [17].

The plant *SnRK* family is widely involved in metabolic regulation, plant growth and progress, and abiotic stress response. *SnRK1* is the main regulator of cellular energy homeostasis, which is primarily involved in metabolic regulation and affects plant development. *SnRK1* can phosphorylate its downstream target, *bZIP63*, and activate the cytoplasmic pyruvate phosphokinase (cyPPDK) promoter, thereby coordinating the metabolic and developmental mechanisms of *A. thaliana* seedling [18]. *A. thaliana SnRK1* (*AtSnRK1)* phosphorylates a *bZIP63* transcription factor that binds directly to the *ARF19* promoter and activates it, thereby regulating lateral root (LR) formation [19]. Among the three *SnRK1* genes in *A. thaliana*, *KIN10* (SNF1 kinase homolog 10)/*SnRK1.1* and *KIN11* (SNF1 kinase homolog 11)/*SnRK1.2* play an imperative role since the expression of *KIN12*/*SnRK1.3* cannot be detected in most tissues [20]. Of these, the *KIN10* protein can interact with the stomatal development core transcription factor SPEECHLESS (SPCH) and phosphorylate SPCH to improve the protein stability of SPCH and thus promote stomatal development [21]. The protein encoded by members of the *SnRK2* subfamily is a monomeric kinase that regulates plant response to various abiotic stresses. Most *SnRK2* can be activated by hypertonic stress, except *AtSnRK2.9* [12, 22]. *SnRK2*-*PYR/PYL/RCARs*-*PP2C* is the core pathway for plant response to ABA [23]. During the process, the SnRK2 in plants is switched from a quiescent dephosphorylated state to an activated state that is the center reaction of plants response to ABA and natural stresses. Studies revealed that in *A. thaliana,* the protein kinase of the B2/3 subset of RAF might phosphorylate and induce *SnRK 2.2/2.3/2.6*. The protein kinase of the B4 subgroup of RAF might phosphorylate and activate the other 6 ABA-independent *SnRK2*. It reveals the central part of the *RAF-SnRK2* cascade pathway in osmotic stress and ABA signaling [2, 3, 24]. ABA-*PYL*-*PP2C* is responsible for inhibiting *SnRK2* binding release, while RAF mediates *SnRK2* self-activation through phosphorylation, thus initiating *SnRK* activation [4]. AtSnRK2 protein kinase could be activated by drought and ABA treatment and phosphorylate sucrose transport proteins SWEET11 and SWEET12, thereby promoting root growth under drought stress, improving root-shoot ratio, and drought resistance [25]. As part of the calcium signaling pathway, *SnRK3* mediates plant development and responses to ionic and nutritional stress such as high sodium levels, potassium deficiency, and nitrogen starvation [5, 26]. When the plant was under high salinity, the generated calcium signal was sensed by *SOS3* (*AtCBL4*) on the cell membrane. Then *SOS3* is combined with *SOS2* (*AtCIPK24*) to form a complex that phosphorylates *SOS1* (Na+/H+ antiporter) and removes the excess Na + from the root cells [27, 28]. The CBL1/9-CIPK23 complex could enhance the absorption of environmental potassium by plant cells by activating the potassium channel *AKT1* [29, 30] and the plasma membrane potassium transporter *HAK5* [31, 32]. In vitro recombination experiments showed that the C*BL2/3-CIPK3/9/23/26* fractions efficiently triggered the activity of *TPK1/ 3/ 5*, which depended on the cytoplasmic calcium concentration [33]. *MdCIPK13* and *MdCIPK22* improve salt and drought tolerance by targeting the phosphorylated sucrose transporter *MdSUT2.2* in apples [27, 34]. Overexpression of *BnCBL1*-*BnCIPK6* in *Brassica napus* enhances tolerance to high salinity and low potassium [35]. In addition, *CIPK14* also regulates the glucose response and interacts with *KIN10*/*SnRK1.1* and *KIN11*/*SnRK1.2* in *A. thaliana* [36]. Furthermore, the upstream activated kinases of *SnRK1*, *SnAK1*/*GRIK1*, and *SnAK2*/*GRIK2* phosphorylate and activate *SOS2* under salt stress [37]. These results indicate an interaction between members of the *SnRK* subfamily, which plays a role in plant physiological processes besides their important role in responding to high salinity and drought stress. However, the representation of *SnRK* family members involved in low-temperature stress response needs further investigation.

As plant genomes are increasingly sequenced and analyzed, the plant *SnRK* protein kinase family has received ceaseless recognition. Its auxiliary properties and expression profiles in response to natural stresses such as salt, drought, and ABA have been explained [38–40]. In this study, members of the *L. chinense* SnRK (LcSnRK*)* protein kinase family were analyzed for their structural properties and expression profiles under low-temperature stress. It is grown in southern china and is a vital ornamental species [41]. The low-temperature response genes of *Liriodendron* are expected to be tapped to provide genetic resources and theoretical foundations for the future improvement of *Liriodendron* germplasm through genetic engineering.

## Results

### Documentation and physicochemical properties analysis of *LcSnRK*

**Fig. 1:**
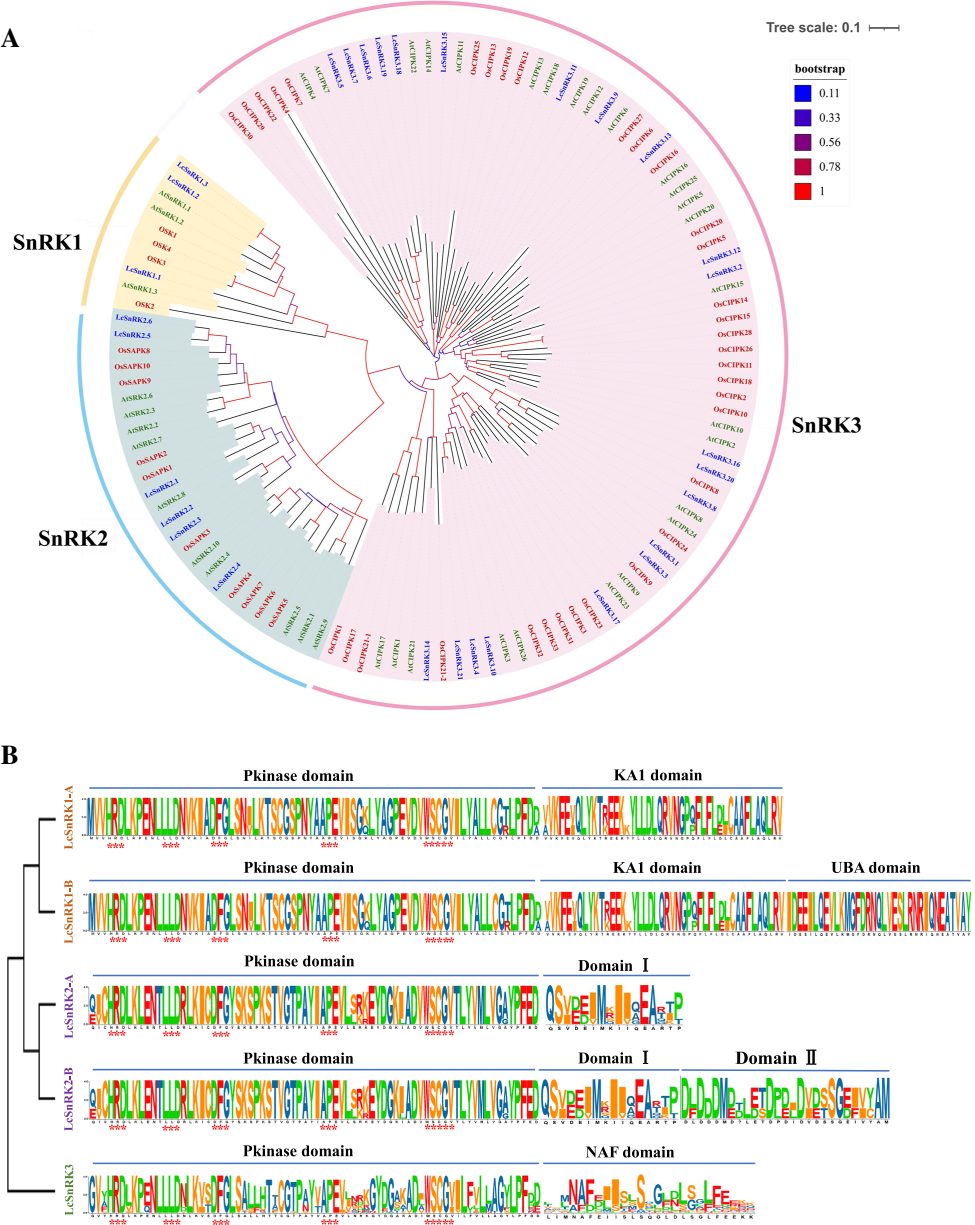
Conserved domains of SnRKs and their phylogenetic relationship. **A** Distribution of conserved domains in three subfamilies of SnRK. **B** Phylogenetic tree of SnRK. Green, red, and blue fonts represent members of the AtSnRK, OsSnRK, and LcSnRK families, respectively. Background yellow, blue, and red areas represent SnRK subfamilies 1, 2, and 3, respectively

In this study, 30 members of *LcSnRKs* were identified (Additional file 1: Table S1). Three members of *LcSnRKs* were categorized into the LcSnRK1 subfamily by Pfam analysis. All delimited the kinase-associated domain KA1 (PF02149), except for two members of LcSnRK1s, which also contained the ubiquitin-related domain (UBA) (PF00627). Therefore, we can further subdivide the members of the LcSnRK1 subfamily without the conservative domain of UBA into SnRK1-A; Members of the LcSnRK1 subfamily with the conservative domain of UBA have been classified as LcSnRK1-B (Fig. 1A). Six *LcSnRKs* were allocated to the SnRK2 subfamily based on the assessment of the conserved domain I, which is approximately 30 amino acids distant from the kinase domain, except for three members of LcSnRK2s that also contained the domain II. Therefore, we can further subdivide the LcSnRK2 subfamily into SnRK2-A and SnRK2-B according to the presence or absence of domain II. Twenty-one LcSnRKs belong to SnRK3/CIPK subfamily, containing the NAF domain (PF03822). This domain comprises 24 amino acids, which can be combined with CBLs. Based on the conventional domain and its arrangement on the chromosome, 30 members of *LcSnRKs* have been named *LcSnRK1.1* ~ *LcSnRK1.3*, *LcSnRK2.1* ~ *LcSnRK2.6*， *LcSnRK3.1* ~ *LcSnRK3.21*. The amino acid number distribution of proteins from *LcSnRK1s*, *LcSnRK2s*, and *LcSnRK3s* ranged between 519 ~ 547aa, 265 ~ 355aa, 356 ~ 629aa, respectively. The molecular weight ranges of the proteins of *LcSnRK1s*, *LcSnRK2s*, and *LcSnRK3s* were 58.36 ~ 62.44 kDa, 29.77 ~ 40.95 kDa, and 39.95 ~ 70.77 kDa, respectively.

Of the average length and molecular weight of the proteins within the three LcSnRK subfamilies, the amino acid number (537.7 aa) and molecular weight (61.08 kDa) of LcSnRK1s were the largest, while the amino acid number (301.17 aa) and the molecular weight (34.15 kDa) of LcSnRK2s were the smallest.

According to the analysis of the instability index (I.I.), the proteins of LcSnRK1s were predicted to be unstable; half of the proteins of LcSnRK2s (LcSnRK2.2/2.3/2.6) were stable; however, most proteins of the LcSnRK3s were stable (among them, only the four proteins of LcSnRK3.8/3.9/3.18/3.19 were unstable). Except for the predicted signal peptide in LcSnRK 3.21 (the signal peptide cleavage site is within amino acids 1–25), most LcSnRK proteins lacked the signal peptide. Subcellular localization prediction suggested that all LcSnRKs proteins were localized in the nucleus (Additional file 1: Table S1).

### Phylogenetic relationship of *SnRK* gene family

To investigate the systemic diversity relationship among altered subfamilies and members of *LcSnRK*, an NJ phylogenetic tree was generated based on *L. chinense* SnRK protein sequences. It was initiated that *LcSnRK* was divided into three subfamilies, *LcSnRK1*, *LcSnRK2*, and *LcSnRK3* (Additional file 2: Fig. S1). Phylogenetic trees constructed using the SnRK family proteins from three species, *A. thaliana*, rice, and *L. chinense*, exhibited a similar number of members in the *SnRK1s* (three to four) and the *SnRK2s* (six to ten) that were present in *L. chinense*, as well as *A. thaliana* and rice. Details of the *SnRK* gene family for rice and *A. thaliana* are presented in Additional file 3: Table S2. Conversely, the number of members of *SnRK3s* varies by species. For example, 26 are found in *A. thaliana*, 34 in rice, and 21 in *L. chinense* (Fig. 1B).

On the phylogenetic tree, the SnRK genes of each group were distributed equally. Members of the rice SnRK family cluster in the evolutionary branches of the phylogenetic trees outer group. The SnRK1 subfamily has many similarities among its members. Among them, three species had bootstrap scores greater than 50, which may reflect the conservative evolutionary position of *LcSnRK1*. The bootstrap values of the branches in the *SnRK2* and *SnRK3* subfamilies are not all greater than 50. Especially in the *SnRK2* subfamily where *LcSnRK2.4* and *AtSnRK2.4* cluster on the same branch; *LcSnRK2.1/2.2/2.3* and *AtSnRK2.7/2.8* were clustered on the same evolutionary branch; *LcSnRK2.5/2.6* and *AtSRK2.2/2.3/2.6* were clustered on the same branch.

### Examination of the gene structure and conserved motif of LcSnRKs

**Fig. 2:**
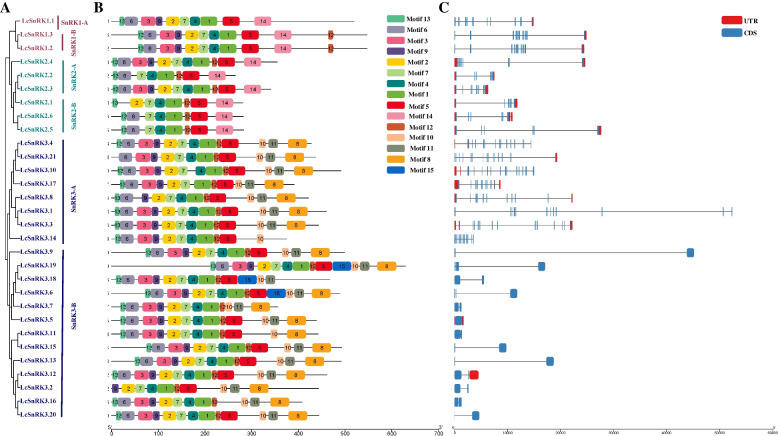
Conservative motif and exon-intron pattern of *Lc*SnRKs. **A** Phylogenetic trees of three subfamilies of LcSnRKs. **B** Conservative motifs of three subfamilies of *Lc*SnRKs. **C** An exon-intron pattern of three subfamilies of *Lc*SnRKs

To explore the development of preparing the *SnRK* family in *L. chinense*, the moderated themes of 30 individuals from the *LcSnRK* family were analyzed in this consideration (Additional file 4: Fig. S2). Dissimilar subfamilies were found to share common features in motif composition and significant differences (Fig. 2A, B). Motifs 1, 4, and 7 encode a pkinase domain in all *LcSnRK* genes. Motif 14 only exists in the LcSnRK1 and LcSnRK2 families. Motif 12 is presented in all members except SnRK1-A. Motif 12 in SnRK1-B is arranged at the N-terminal. The arrangement pattern of motifs 12, 5, and 14 only exists in the SnRK2 subfamily. Motifs 10, 11, and 8 encode the NAF conservative domain, so these three motifs only exist in the SnRK3 subfamily. In addition, members of the same subfamily shared similar features in the exon-intron structures of the *LcSnRK* family genes (Fig. 2A, C). *LcSnRK1s* contain 10 to 11 introns; *LcSnRK2s* contain 6 to 9 introns. In contrast, the number of introns varies in the *LcSnRK3* subfamily, with 13 members containing three or fewer introns and 8 members containing over 10 introns, which differ from the other two subfamilies. Therefore, *LcSnRK3* could be divided into two subgroups according to the number of introns, an intron-rich subgroup, and an intron-deficient subgroup. The characteristics of the number of introns suggest that genes in the *SnRK1* and *SnRK2* subfamilies are structurally more conservative than genes in the *LcSnRK3* subfamily.

### Cis-regulation element of *LcSnRKs*

**Fig. 3:**
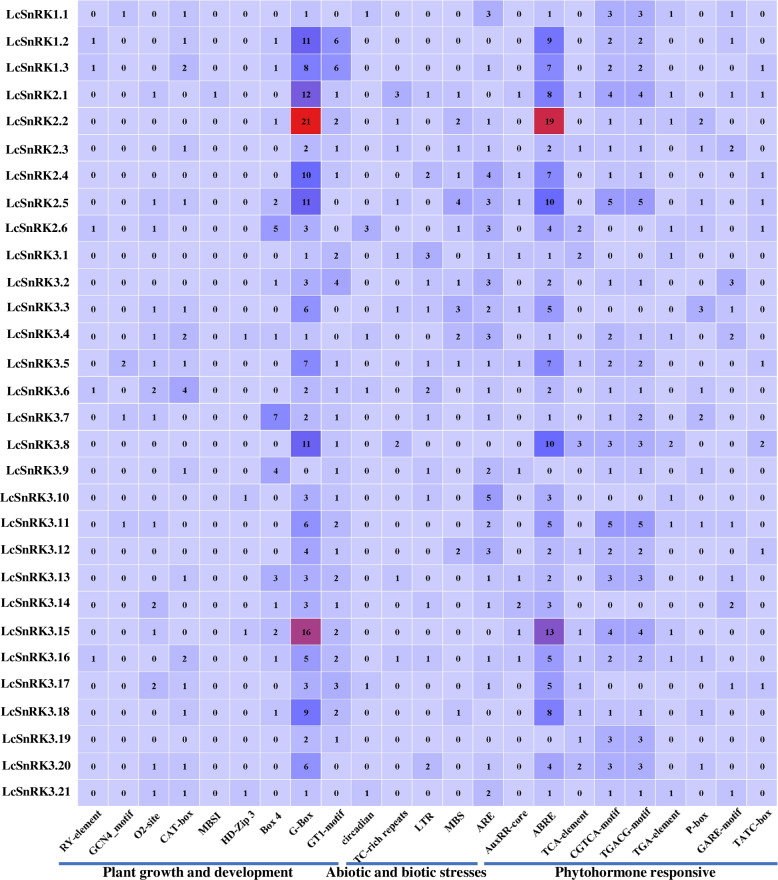
Typical cis-regulation element analysis of *LcSnRKs*

Considering that the *SnRK* gene family plays a critical part in many natural forms, the promoter of the *LcSnRK* gene was analyzed in this study to recognize potential administrative components. Hence, giving clues for a more profound understanding of quality control and its reactions provides distinctive boosts. We analyzed the cis-regulatory elements (CREs) on 2 kb of the putative promoters of the *LcSnRK* gene family members. The detailed distribution of each cis-acting element in the promoter region is presented in Additional file 5: Fig. S3. Here, we grouped the CREs according to response to abiotic and biotic stresses, response to phytohormones, and plant growth and development. The heatmap indicates whether the various elements are over- or under-represented relative to 30 members of the *LcSnRK* family (Fig. 3). G-Box and ABRE are the most widely distributed, and *LcSnRK2.2* has the most G-Box and ABRE elements. In the *LcSnRK1* subfamily and the *LcSnRK2* subfamily, most members (*LcSnRK1.2*/*1.3*/*2.1*/*2.2*/*2.4*/*2.5*) have more G-Box and ABRE elements (over six). Only four members of the *LcSnRK3* subfamily (*LcSnRK3.5/3.8/3.15/3.18*) had more G-Box and ABRE elements. The 12 *LcSnRK* genes contained LTR, a cis-acting element associated with a low temperature. *LcSnRK3.1* contained three LTRs, and the *LcSnRK1* subfamily contained no low-temperature responsive elements. Jasmonates (JA) are involved in various aspects of plant development and are part of the growth-defense trade-off. The 22 *LcSnRK* genes contained methyl jasmonate-related regulatory elements, including the CGTCA-motif and TGACG-motif. *LcSnRK2.5* and *LcSnRK3.11* contained most of the CGTCA- and TGACG-motif elements, which are over-represented on the *LcSnRK* promoters.

### Chromosomal location, genomic collinearity, and gene duplication analysis of *LcSnRKs*

**Fig. 4:**
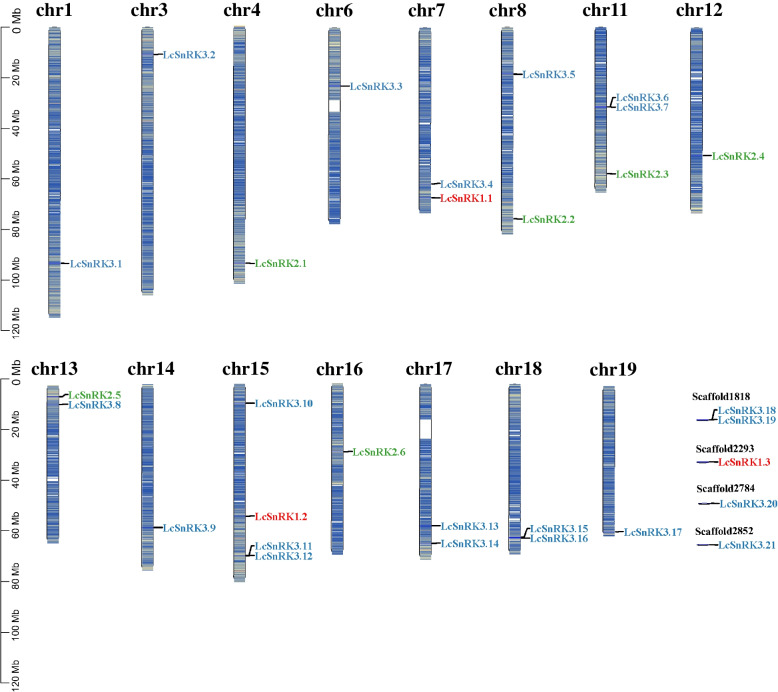
Chromosomal distributions of *LcSnRK*s. Gene density is shown on each chromosome. The members of *LcSnRK1* subfamily are represented in red, the members of *LcSnRK2* subfamily in green, and the *LcSnRK3* subfamily in blue

**Fig. 5:**
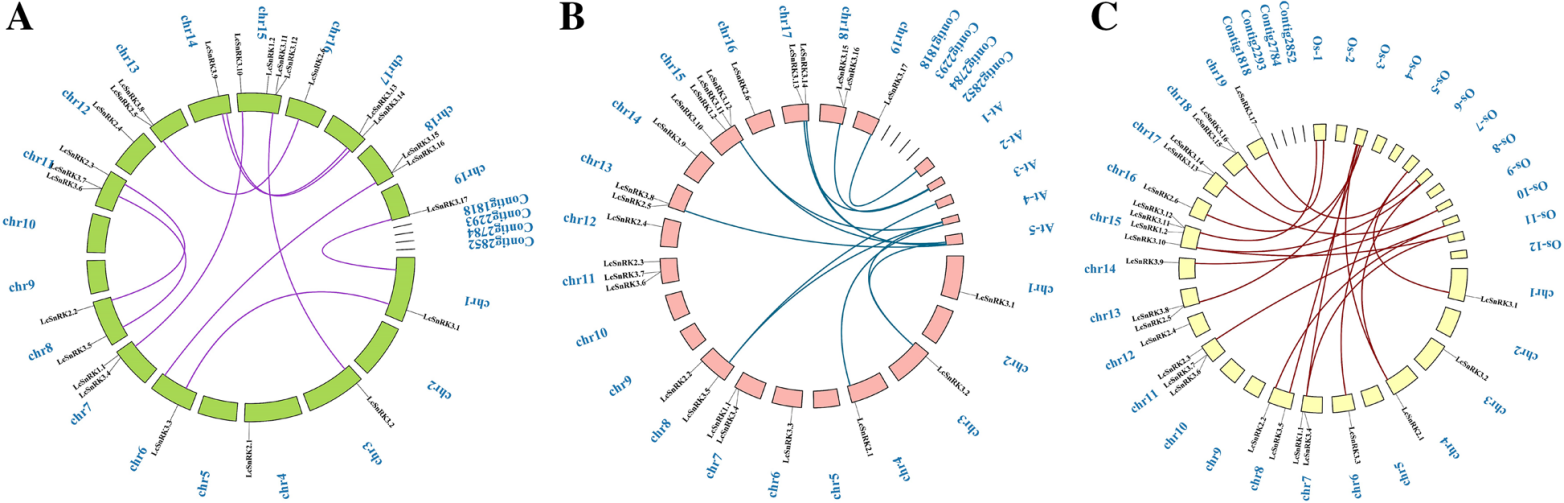
**A** Segment duplicate pairs of *LcSnRKs*. **B** Collinear gene pairs between the SnRK family of *Liriodendron chinense* and *Arabidopsis thaliana*. **C** Collinear gene pairs between the SnRK family of *Liriodendron chinense* and rice

Twenty-five members of the 30 *LcSnRK* genes were distributed across 16 chromosomes, and 5 genes (*LcSnRK1.3*, *LcSnRK3.18*, *LcSnRK3.19*, *LcSnRK3.20*, *LcSnRK3.21*) were mapped to non-assembled genomic fragments (Fig. 4). The positions of the *LcSnRK* family genes on the chromosomes were relatively dispersed. Four LcSnRK3 subfamily groups have been arranged into clusters on various chromosomes, including LcSnRK3.6/LcSnRK3.7, LcSnRK3.11/LcSnRK3.12, LcSnRK3.15/LcSnRK3.16, and LcSnRK3.18/LcSnRK3.19. These four gene clusters are called tandem duplicates, in which the two 200 bp fragments produced by duplication are within the same chromosome. The term “segment duplication” refers to the occurrence of two duplicate gene pairs in which fragments are on different chromosomes or are dispersed far apart on the same chromosome [42]. Based on BLAST and MCScanX, 10 segment duplication events were identified in this study, and all occurred between different chromosomes (Fig. 5A). This result demonstrated that segment duplication occasions are vital in enhancing the *LcSnRK* gene. No duplication events were observed within the *LcSnRK1* subfamily, affirming the elevated level of preservation within the *LcSnRK1* subfamily.

To explore the homology of the *SnRK* gene family in *L. chinense* with that in other species, the homology-collinearity analysis of the *SnRK* gene in *L. chinense*, *A. thaliana,* and rice was performed in this study (Fig. 5). The result showed that 13 *LcSnRK* genes and *AtSnRK* genes were in a collinear relationship (Fig. 5B), and 18 *LcSnRK* genes were collinear with *Oryza sativa SnRK (OsSnRK)* genes (Fig. 5C). Therefore, it has been suggested that these *LcSnRK* genes might be involved in the evolution of the *LcSnRK* gene family. To test the effect of evolutionary constraints, Ka, Ks, and Ka/Ks of paralytic homologous and orthologous pairs on the *SnRK* gene family were calculated. The Ka/Ks range for the 10 segment-duplicated gene pairs was 0.055 to 0.295 (Additional file 6: Table S3), indicating that all duplicated gene pairs for *LcSnRK* were purified by selection. The Ks of the 13 homologous gene pairs in *L. chinense* and *A. thaliana* were all “NaN”, indicating that the sequences of the gene pairs in *L. chinense* and *A. thaliana* had a high score of divergences (Additional file 7: Table S4). The Ka/Ks of the homologous gene pairs in most *L. chinense* and rice were also much smaller than 1 (Additional file 8: Table S5), suggesting that the *LcSnRK* gene family may have arisen during evolution by purifying selection.

### Protein interaction of SnRKs

**Fig. 6:**
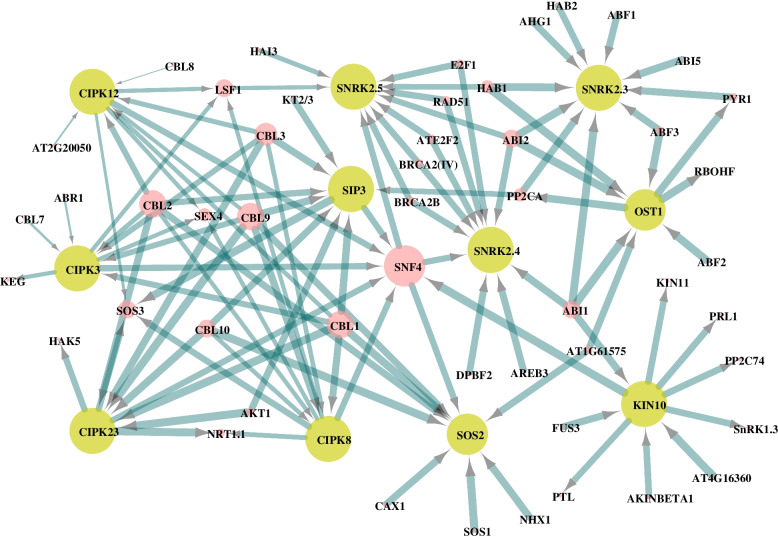
Protein interaction analysis of *SnRKs* and other proteins. The yellow circles represent the hub gene in the protein-protein interaction network

Exploring the regulatory network of a gene or a gene family is essential to restore the balance between resistance and growth. Therefore, the interaction network of the LcSnRK protein was analyzed based on its homology with *A. thaliana* (Fig. 6, Additional file 9: Table S6). In the protein-protein interaction network, it was found that KIN10, SnRK2.3, SnRK2.4, SnRK2.5, OST1, CIPK3, CIPK8, SOS2, CIPK12, SIP3, and CIPK23 existed at the center of the interaction network and served as an important hub, which might play a central role in life activities. In the SnRK1 subfamily, KIN10 can interact with the transcription factor FUSCA3 (FUS3) and some disease-causing related genes *PRL*. There was also an interaction between three members of this SnRK1 subfamily.

In the SnRK2 subfamily, members of SnRK2 can interact with ABI1; SnRK 2.3/SnRK2.4/SnRK2.5/SnRK2.6 (OST1) can interact with ABI2; In addition, SnRK2.3 can interact with ABI5, suggesting that the ABI family may be closely related to the SnRK2 subfamily. Both SnRK2.4 and SnRK2.5 can interact with two members of the BRCA2 family.

Within the SnRK3 subfamily, a common SOS signaling pathway and a CIPK-CBL complex pathway like the CBL3 (calcineurin B-like protein 3) have been associated with CIPK3, CIPK8, CIPK23, CIPK12, and SIP3. CIPK23 and SIP3 can also activate AKT1 on the potassium channel. All three subfamilies of SnRK can interact with the same gene family (e.g., the PP2C protein phosphatase family). There are also interactions between the three subfamilies of SnRK. For example, ABI1 can simultaneously bind to and interact with KIN10 and OST1, affecting the expression of downstream genes (Fig. 6, Additional file 9: Table S6).

### Three-dimensional structures of SnRK proteins

**Fig. 7:**
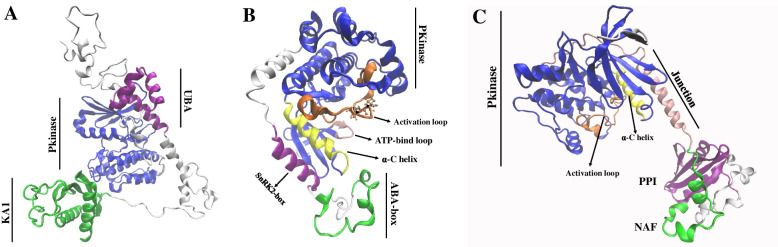
Three-dimensional structure model of *LcSnRK1.3* (**A**), *LcSnRK2.3* (**B**), *LcSnRK3.1* (**C**)

Kinases are widely involved in plant signal transduction in response to various types of stress, including the SnRK gene family. Based on previous studies, some key members of the SnRK family, such as KIN10, OST1, and SOS2, play important roles in the stress response. LcSnRK1.3, LcSnRK2.3, and LcSnRK3.1 showed the highest homology to KIN10, OST1, and SOS2, respectively, and were selected to analyze their three-dimensional structures (Additional file 10: Table S7). From the three-dimensional structure of LcSnRK1.3, it contained a kinase domain and a UBA domain (ubiquitin-related domain), and LcSnRK1.3 also contained the KA1 domain at the C-terminus (Fig. 7A; Additional file 11: Fig. S4).

Further modeling and comparing the spatial structure of the LcSnRK1.3 catalytic domain confirmed the significant similarity in the three-dimensional structure between LcSnRK1.3 and KIN10. SnRK2.6/OST1 (Open Stomata 1) was crucial for ABA-induced stomatal closure in response to drought. LcSnRK2.3 contains a conservative kinase domain and a C-terminal regulatory region containing two conservative motifs that form the SnRK2 box. The SnRK2 box forms a single α-helix and runs parallel to the αc-helix (Fig. 7B; Additional file 12: Fig. S5). In addition, like AtOST1, LcSnRK2.3 contains the auto-phosphorylated residues S175 and T176. The highest homology (74%) was found between LcSnRK3.1 and AtSOS2 (Fig. 7C; Additional file 13: Fig. S6). Typical catalytic domains of CIPKs were found in LcSnRK3.1, including an N-terminal kinase catalytic domain, a self-inhibitory motif characteristic of NAF, and a protein kinase 2C-binding domain of PPI. The three-dimensional structure of LcSnRK3.1, which activated the ring of LcSnRK3.1 from its active site, was remarkably similar to AtSOS2.

### Expressions of *LcSnRKs* in response to low-temperature stress

**Fig. 8:**
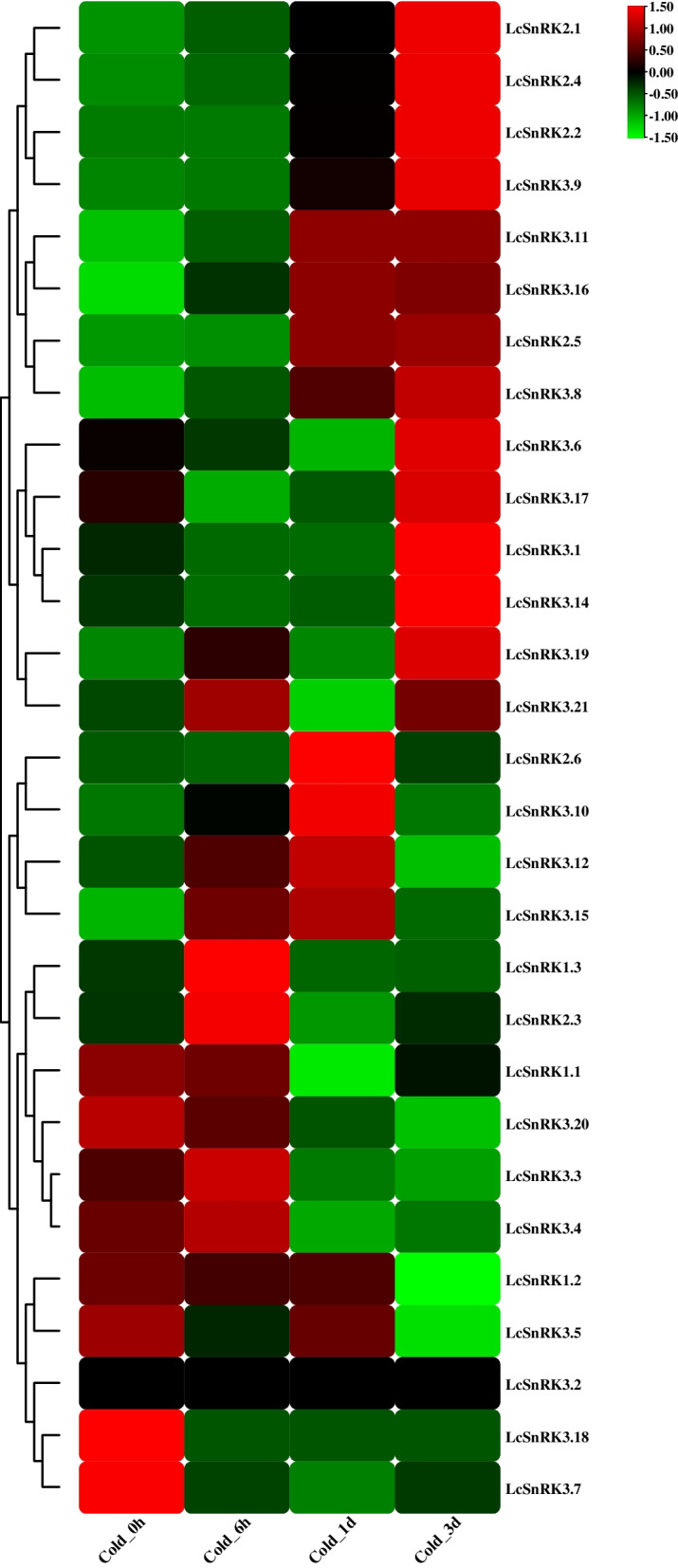
RNA-seq expression pattern of *LcSnRK* family genes under low-temperature stress

To understand the responses of LcSnRK family individuals to low-temperature stress, transcriptome information from *L. chinense* elevating under low-temperature stress was selected to analyze the expression design of the *LcSnRK* quality under low-temperature response (Fig. 8). The low-temperature stress treatment cycle could be roughly divided into continuous up and down-regulation. Expression of 29 *LcSnRK* genes was detected in the *L. chinense* cryo transcriptome data, except for the lack of expression of *LcSnRK3.2*. Four genes of the *LcSnRK2* subfamily (*LcSnRK2.1/2.2/2.4/2.5*) and nine genes of the *LcSnRK3* subfamily (*LcSnRK3.1/3.6/3.8/3.9/3.11/3.14/3.16/3.17/3.19*) were continuously up-regulated. The expression of eight genes (*LcSnRK2.1/2.2/2.4/2.5/3.8/3.9/3.11/3.16*) increased after 1 day of low temperature stress. The expression of five genes (*LcSnRK3.1/3.6/3.14/3.17/3.19*) increased after 3 days of low-temperature stress. One gene in the *LcSnRK1* subfamily (*LcSnRK1.1*) and five genes in the *LcSnRK3* subfamily (*LcSnRK3.3/3.4/3.7/3.18/3.20*) were continuously down-regulated.

Among them, the expression of four genes decreased after 6 hours of low-temperature stress (*LcSnRK1.1/3.7/3.18/3.20*), and the expression of two genes decreased after 1 day of low-temperature stress (*LcSnRK3.3/3.4*). Six genes (*LcSnRK1.3/2.3/2.6/3.10/3.12/3.15*) were briefly up-and down-regulated, and two genes (*LcSnRK1.3/2.3*) indicated the highest expression level after 6 hours of low-temperature treatment, followed by the weakness in the expression level.

Four genes (*LcSnRK2.6/3.10/3.12/3.15*) showed the highest expression level 1 day after low-temperature treatment, while their expression level decreased 3 days later. *LcSnRK1.2* expression was stable within 1 day and declined abruptly after 3 days. *LcSnRK3.5* expression decreased during the first 6 hours of low-temperature treatment, increased after 6 hours, and decreased to a minimum after 3 days.

**Fig. 9:**
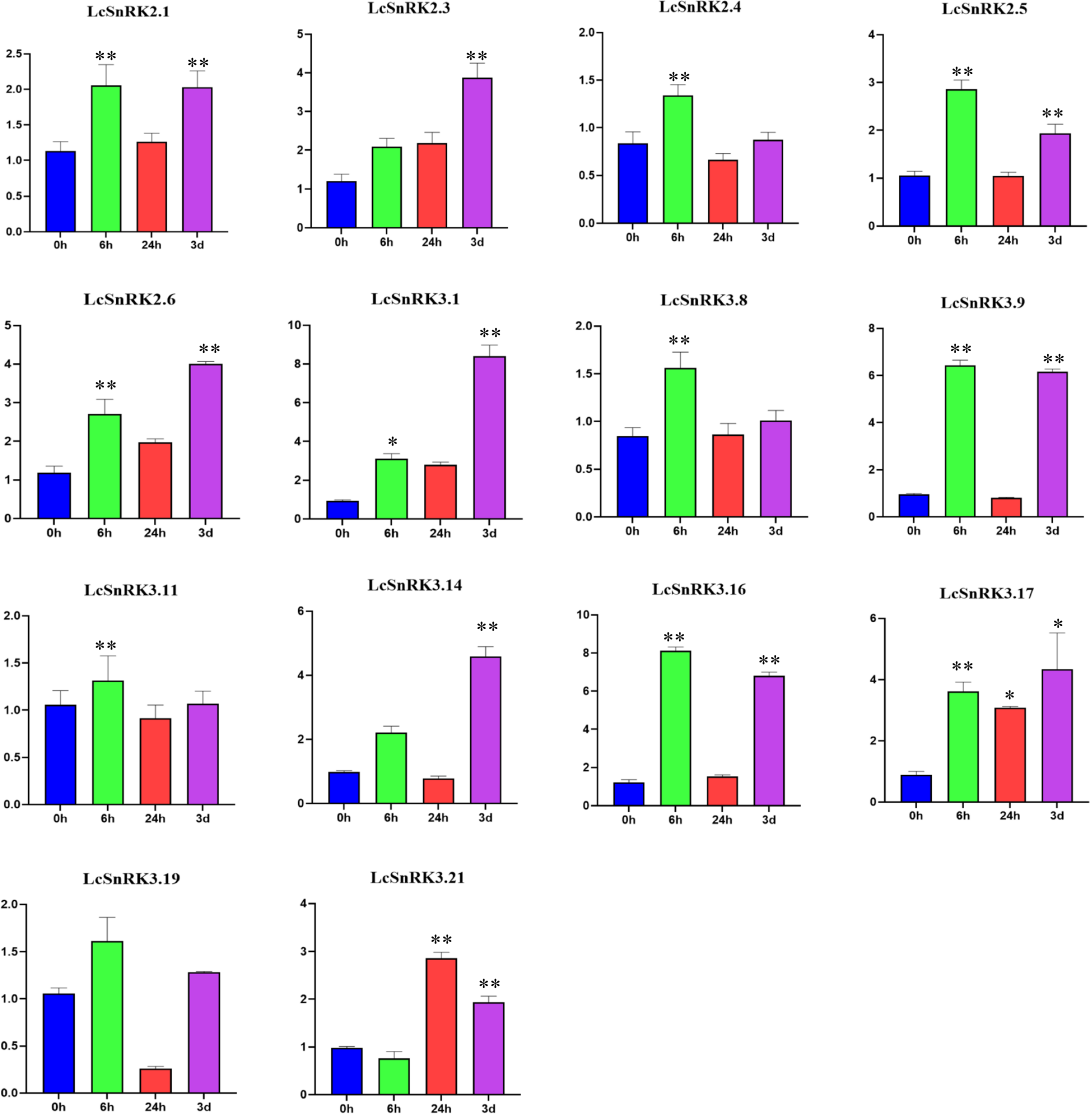
Expression patterns of qPCR of *LcSnRK* family genes under low-temperature stress. Y-axis represents relative expression, and X-axis represents different time points after stress treatment for expression analysis; The mean standard error measurement (SEM) values of three replicates (*n* = 3) are shown. Each time point was compared with the control group, and the significance was calculated by one-way ANOVA: * means 0.0001 < *p* < 0.0005, and ** means *p* < 0.0001

To further affirm the expression design of the *LcSnRK* gene within the over transcriptome investigation, five genes of the *LcSnRK2* subfamily (*LcSnRK2.1/2.3/2.4/2.5/2.6*) and nine genes of the *LcSnRK3* subfamily (*LcSnRK3.1/3.8/3.9/3.11/3.14/3.16/3.17/3.19/3.21*) were chosen for qPCR confirmation. qPCR and RNA-seq were found to have similar expression patterns (Fig. 9). The transcriptome expression of these 14 *LcSnRK* genes and the expression trend of qPCR were up-regulated, and the expression trend of eight genes (*LcSnRK2.1/2.4/2.5/3.8/3.9/3.11/3.14/3.16*) in the transcriptome was continuously up-regulated, with a wavelike increase in qPCR. Five genes (*LcSnRK2.6/3.1/3.17/3.19/3.21*) indicated wavy up-regulation in both transcriptome and qPCR. One gene (*LcSnRK2.3*) showed a wavelike up-regulation in the transcriptome, and expression in qPCR continued to increase.

## Discussion

The SnRK family plays a vital part in plant response reaction signaling pathways and is a keeper in all eukaryotes. *SnRK1* is involved in cell energy sensing, while *SnRK2* and *SnRK3* play fundamental roles in signaling pathways and regulation of gene expression [43, 44]. In later years, different capacities of *SnRK* family qualities in plants were slowly found and presented in plants [18, 45–47], suggesting that the *SnRK* gene has greater importance in plant life activities. In this research, a total of 30 members of the *LcSnRK* family were identified from the *L. chinense* genome. All *LcSnRK* members contain kinase domains classified according to their different domains and functions as members of the *LcSnRK1*, *LcSnRK2*, and *LcSnRK3* subfamilies. The six members were classified into the *LcSnRK2* subfamily, and *LcSnRK2* could be further classified into three classes according to protein sequence and function. This corresponded to the classification of the *SnRK2* subfamily in *A. thaliana* [11, 13, 48]. While the first group of *SnRK2s* (*AtSnRK*2.1/4/5/9/10) revealed no effects by ABA, the second group of *SnRK2s* (*AtSnRK*2.7/8) revealed fewer effects by ABA, and the third group of *SnRK2s* (*AtSnRK*2.2/3/6) revealed highly activated by ABA. Complete activation of *OST1* (the highly homologous kinase of *LcSnRK2.3*) requires a closed conformation of the αC-helix and phosphorylation of the activation loop to allow complete alignment of the catalytic residues in the active conformation. A highly acidic ABA box, an unknown mechanism required for kinase activity, is important for mediating the interaction of SnRK2 with protein phosphatase type 2C (PP2Cs) [22, 49]. S175 and T176 in *OST1* face the catalytic site and thus provide the basis for efficient autophosphorylation of *OST1*, leading to its full activation [50]. *LcSnRK3* shares the highest homology with *SOS2* among its 21 family members. Recent studies have shown that *CIPK24* (*SOS2*) has basal activity, unlike other members of the *CIPK* family. At the substrate, the active loop of *SOS2* protrudes from the active site, allowing for catalysis. These and other data suggest that securing *CIPKs* kinase activity requires simultaneous release of the activation loop from the active site and the NAF motif from the nucleotide-binding site [51]. This structure suggests that *LcSnRK3.1* is not a self-inhibitory authoritative versicle for *CIPKs* and represents a compositional highlight of the environment, suggesting that plant homologs may be involved in controlling cytoskeletal structure and function.

The SnRK proteins from rice, *A. thaliana*, and *L. chinense* were developed on the same phylogenetic tree using the neighbor-joining strategy to analyze their origin and characteristics. The *LcSnRKs* were divided into three subgroups and clustered with the corresponding subgroups of rice and *A. thaliana* during phylogeny. Therefore, three *SnRK* subgroups must be established before the diversity of dicotyledonous and monocotyledonous plants. Conservative structural motif analysis provided information on the conservation of LcSnRK function during evolution. We found five conservative motifs related to the functional domain in the plant SnRK protein. Motif 2 and 4 contained Ser/Thr active site and ATP-binding region, respectively. Motif 12, motif 14, and motif 8 contain specific conservative domains of SnRK1 (KA1), SNRK23 (glutamine − 303 to proline − 318), and SnRK3 (NAF), respectively. Different patterns of exon-intron structures also play an essential role in the evolution and function of different gene families [52]. *SnRK3* subfamily differs in gene number and can divide *SnRK3* subfamily into two branches according to the number of introns [53, 54].

Similarly, the *LcSnRK3* subfamily can be divided into two branches: intron-rich and intron-poor. In rice and *A. thaliana*, the *SnRK3* subfamily was subdivided according to the number of introns, suggesting that an increase or decrease in the number of introns might promote the structural evolution of the *SnRK* gene family prior to eudicots-monocots differentiation [54]. Colina et al. [44] found that the *SnRK* family originates from green algae, and almost all members respond to osmotic stress. The intron-poor group first appeared in the seed plants. If seed plants were subjected to huge natural weights amid advancement, the intron-rich population would lose introns and become an intron-less population [44]. Similar exon-intron patterns and arrangements of conservative motifs indicate a close evolutionary relationship within the same subfamily. In addition, Jeffares et al. [55] found that the density of introns of genes that can be rapidly expressed in stress responses is significantly lower. Liu et al. [56] speculated that intron loss might be an evolution of rapid stress adaptation in the early evolution of land plants. They also suggested that the absence of introns may have facilitated more rapid developmental adaptation in terrestrial organisms. Therefore, it is reasonable to speculate that genes in the intron deletion group in the *SnRK3* subfamily become more highly expressed under stress.

Genomic duplication occurs throughout plant advancement and often leads to the extension of gene families [57]. The evolution of angiosperms frequently exhibits two major types of gene duplication patterns known as tandem and segment gene duplication [58] and plays a significant role in gene family expansion [59]. This study found 14 homologous gene pairs within the *LcSnRK* family, counting 10 sets of segment-duplicate genes and 4 sets of gene clusters. In addition, we identified 13 pairs of homologous gene pairs between *L. chinense* and *A. thaliana* and 18 pairs between *L. chinense* and rice. The *SnRK2* and *SnRK3* subfamilies are plant-specific and arise through duplication of the *SnRK1* family [43, 44]. The expansion of the *SnRK* family is likely because large plants are sessile organisms subject to greater biotic and abiotic pressures than animals [44]. The Ka/Ks ratio of the full-length encoded protein sequences of 14 pairs from collateral homologous gene pairs was much lower than 1, indicating that most genes were effectively purified and selected.

Tissue- and time-specific expression patterns of genes in growing plants often reflect differences in the biological function of gene family members and interactions between related signaling pathways [60, 61]. At the transcriptional level, a total of 21 genes in the *LcSnRK* family continuously increased or decreased under low-temperature stress. The expression of one member of the *LcSnRK1* subfamily decreased with increasing low-temperature stress, while that of four members of the *LcSnRK2* subfamily increased with increasing low-temperature stress. The expression of 14 genes of the *LcSnRK3* subfamily increased or decreased with increasing low-temperature stress. Regarding evolutionary position, *LcSnRK2.5* and *LcSnRK2.6* were the closest to *OsSAPK8* in rice (Fig. 1B). The rice ossapk8 mutant exhibited slow growth and yellowing of leaves under low temperature treatment [62]. Moreover, from the expression pattern of *L. chinense* leaves under low temperature treatment, the expression of *LcSnRK2.5* reached its highest at 6 hours after low-temperature treatment. It then declined but rose 3 days later. The expression of *LcSnRK2.6* reached its peak at 3 days of low temperature treatment. In addition, when *Agropyron cristatum AcSnRK2.11* was transferred to tobacco, the transgenic plants indicated stronger cold resistance [63]. *LcSnRK3.5/3.6/3.7/3.19* is closer to *AtCIPK7* in evolutionary terms (Fig. 1B). In *A. thaliana*, *CIPK7* can interact with *CBL1* in vivo and in vitro to play a role in the low-temperature response [64]. Under low-temperature treatment, the expression of *LcSnRK3.19* reached its peak 6 hours after low-temperature treatment and then declined but increased after 3 days. In addition, *AcCIPK5*, a cognate gene of *AtCIPK12*, positively regulated the cold tolerance of *A. thaliana* in *Ananas comosus (L.)* [65]. Consequently, we can conclude that individuals of the *SnRK2* and *SnRK3* subfamilies are more likely to be interested in the low-temperature response reaction in *L. chinense*.

Similarly, in the promoter sequence of the *LcSnRK* gene, many cis-acting elements associated with low temperature and other abiotic stresses, such as LTR and TC-rich repeats, were found. These results indicated that the *LcSnRK* gene might respond to abiotic stress, particularly low-temperature stress. In addition, *LcSnRK1.3*, *LcSnRK2.3*, and *LcSnRK3.1* shared high homology with *KIN10*, *OST1*, and *SOS2*, respectively, in *A. thaliana*. The studies on *KIN10*, *OST1*, and *SOS2* in *A. thaliana* were in-depth [1, 66, 67]. *KIN10* is the central integron of the transcriptional network involved in stress and energy signal transduction in plants [1, 68, 69], which can be associated with other family proteins in reaction to abiotic response in plants. *OST1* may be involved in the signaling pathway in plants in response to abiotic stress through phosphorylation [70, 71]. The CBL-CIPK (SnRK3) complex is involved in the signal transduction of various stresses. *SOS2* physically interacts with the calcium sensor *SOS3* [72] and is activated in a calcium-dependent manner [73]. Therefore, the expression of these three genes under abiotic stress indicates certain regularity based on the transcriptional level expression results. For case, *LcSnRK1.1* expression was persistently up-regulated under low-temperature stress, and *LcSnRK2.3* expression appeared to be a short-term expanding drift under low-temperature stress. *LcSnRK3.1* expression was ceaselessly up-regulated under low-temperature response. These three genes may be essential in studying the *L. chinense* response to low-temperature stress.

## Conclusions

The genes of the SnRK family play essential roles in signaling pathways such as plant response to biotic and abiotic stresses. In this study, 30 members of the *SnRK* gene family were identified in *L. chinense* and divided into three typical subfamilies with the same features and obvious differences in gene structure and motif composition. In response to *L. chinense* to low-temperature stress, the 30 members of the *SnRK* gene family had four expression patterns, and the expression trend of 14 genes was continuously increased while that of 6 genes was decreased. These results will give imperative data for examining the structural characteristics and potential natural capacities of the *LcSnRK* gene in *L. chinense*.

## Materials and methods

### Documentation and physicochemical properties analysis of *LcSnRKs*

The nucleic acid and protein sequences of *L. chinense* were obtained from the *L. chinense* protein database (https://hardwoodgenomics.org/) [74]. Protein sequences from the SnRK family of *A. thaliana* and rice were downloaded from the Phytozome database (https://phytozome-next.jgi.doe.gov/) [75], and the rice annotation project (RAP) (https://rapdb.dna.affrc.go.jp/) [76], respectively. Local BLASTP searches were completed using the SnRK protein sequences from *A. thaliana* and rice. The Hidden Markov Model (HMM) and the BLASTP program were used for the preliminary identification of LcSnRK family proteins. LcSnRK candidate proteins were further validated by the NCBI conservative domain database, the SMART database, and the Pfam database (http://pfam.xfam.org/) for domain search [77]. Based on the information in the above three databases, the *LcSnRK* gene with the traditional domain was manually screened. The online software Protparam in the Expasy database (https://web.expasy.org/protparam/) was used to identify sequences of *SnRK* of *L. chinense*, the physicochemical properties (including molecular weight, isoelectric point, and hydrophilicity) [78]. Signal peptides of the LcSnRK protein were predicted using SignalP (https://services.healthtech.dtu.dk/service.php?SignalP). Subcellular localization of the LcSnRK protein was predicted by Plant-mPLoc (http://www.csbio.sjtu.edu.cn/bioinf/plant-multi/) [79].

### Phylogenetic analysis of *LcSnRKs*

Phylogenetic analysis was performed using the *SnRK* members from *L. chinense*, Rice, and *A. thaliana*. The multiple sequences were aligned using ClustalX. The phylogenetic tree was assembled using the NJ method in the MEGA7.0 software [80]. Evolutionary distances were obtained using the p-distance method, which was used to estimate the number of amino acids in each locus. One thousand bootstrap sampling iterations guaranteed the reliability of each phylogenetic tree. iTOL was used for further beautification.

### Chromosome location and gene duplication analysis of *LcSnRKs*

According to the physical location information in the *L. chinense* genome database, the identified *LcSnRK* gene was chromosomally localized using the biological software TBtools [81]. To analyze the duplication events of the *LcSnRK* gene, all *L. chinense* genomic sequences were aligned with BLASTP. The duplication modes of *SnRK* were divided into segment duplication and tandem duplication using MCScanX [82]. Finally, the collinear analysis diagram was constructed using the Circos software [83]. Nonsynonymous (Ka) and synonymous (Ks) substitutions between pairs of interest were calculated using the KaKs_Calculator [84].

### Gene structure, conservative motif, and cis-regulation elements of *LcSnRKs*

The intron-exon structures of the *LcSnRKs* were mapped onto the diagrams using the annotation files in the *L. chinense* genome database by TBtools software. The conserved motifs of the *SnRK* protein in *L. chinense* were analyzed online by MEME (https://meme-suite.org/meme/doc/meme.html) using the predicted full-length protein sequences of the *LcSnRKs*. Extracting an upstream sequence (2000 bp) of an initiation codon of each *LcSnRK* gene from *L. chinense* genomic sequence. PlantCare (http://bioinformatics.psb.ugent.be/webtools/plantcare/html/) [85] was then used to predict the distribution of cis-acting elements in the promoter region of *LcSnRKs*.

### Protein-protein interaction network prediction of SnRKs

Based on the high homology between LcSnRKs and AtSnRKs proteins, a String database (https://cn.string-db.org/) was used to generate a functional protein interaction network. The protein interaction network of the SnRK family was plotted using Cytoscape 3.8.2 [86].

### Three-dimensional structure modeling and verification of LcSnRK proteins

The full-length atomic structure of the LcSnRK protein was assembled by the Robetta program (https://robetta.bakerlab.org/) to construct the structure of the protein. The structure of the candidate protein was further explored since the reliability of its protein structure was determined using the online website SAVESv6.0 (https://saves.mbi.ucla.edu/) including ERRAT, PROVE, Ramachandran. Then the 3D modeling was performed using VMD software.

### Expression analysis of *LcSnRKs* based on transcriptome and qRT-PCR

The expression of LcSnRK in response to cold stress was analyzed using the methods of Guan et al. [87] and Li et al. [88] and momentarily attuned. Two-month-old and identically growing Liriodendron hybrid seedlings regenerated from somatic embryos were transferred to an incubator (25 °C, 16 h light, and 8 h dark) for culture for 1 week and then transferred to a 4 °C incubator for low-temperature stress treatment. Leaves were detached at 0 h, 6 h, 24 h, and 3 days after cold stress treatment for transcriptome sequencing and qRT-PCR analysis. The RNA was extracted using FastPure® Plant Total RNA Isolation Kit (Polysaccharides&Polyphenolics-rich) (Vazyme, Nanjing, China). RNA purity was checked using the NanoPhotometer® spectrophotometer (IMPLEN, CA, USA). RNA integrity was assessed using the RNA Nano 6000 Assay Kit of the Bioanalyzer 2100 system (Agilent Technologies, CA, USA). The library preparations were sequenced on an Illumina Novaseq 6000 platform, and 150 bp paired-end reads were generated [89]. Three replicates were set for each independent experiment. The housekeeping *L. chinense* 18S gene was used as an internal control. All qRT-PCR primers were designed by Primer5.0 (Additional file 14: Table S8). All data generated from real-time PCR amplification was analyzed using a 2^−△△CT^ method.

## Supplementary Information

**Additional file 1: Table S1.** Basic protein information of LcSnRK family members.

**Additional file 2: Fig. S1. **Phylogenetic tree of *LcSnRKs*. The scale of the length of each branch is labeled at the bottom of the graph, and the bootstrap value of each branch is labeled at the node position.

**Additional file 3: Table S2.** Details of the SnRK gene family for rice and Arabidopsis.

**Additional file 4: Fig. S2.** 15 Conservative motifs logo of *LcSnRKs*.

**Additional file 5: Fig. S3.** The detailed distribution of each cis-acting element in the promoter region.

**Additional file 6: Table S3.** The segmental and tandem duplication events of LcSnRKs.

**Additional file 7: Table S4.** The Ka/Ks ratios between *L.chinenese* and *Arabidopsis thaliana*.

**Additional file 8: Table S5.** The Ka/Ks ratios between *L.chinenese* and *Oryza sativa*.

**Additional file 9: Table S6.** Detailed information of interaction network of LcSnRKs with other proteins.

**Additional file 10: Table S7.** Alignment of protein sequences of LcSnRK and AtSnRK family members.

**Additional file 11: Fig. S4.** Sequence alignment of AKIN10 and LcSnRK1.3 protein. Conservative sites in the activation loop are indicated by a red asterisk, and UBA and KA1 are conservative domains in the SnRK1 subfamily, respectively. Differently colored line segments highlight completely conservative and potential phosphorylated residues.

**Additional file 12: Fig. S5.** Sequence alignment of AtOST1 and LcSnRK2.3 protein. Conservative sites in the activation loop are indicated by a red asterisk. Differently colored line segments highlight completely conservative and potential phosphorylated residues.

**Additional file 13: Fig. S6.** Sequence alignment of AtSOS2 and LcSnRK3.1 proteins. Differently colored line segments highlight completely conservative and potential phosphorylated residues.

**Additional file 14: Table S8.** The primers used in the qRT-PCR

## Data Availability

The datasets generated and/or analysed during the current study are available in the [NCBI website] repository, [https://www.ncbi.nlm.nih.gov/assembly/GCA_003013855.2]. The qRT-PCR data supporting the gene relative expression results of this study can be found in Additional file 14. The transcriptome data used in this study has been archived and can also be obtained on the NCBI website. The cold stress accession numbers were PRJNA679089 (https://www.ncbi.nlm.nih.gov/bioproject/PRJNA679089/).

## Abbreviations

SnRK: The sucrose non-fermenting 1 (SNF1)-related protein kinases
KA1: Kinase associated-1 domain
UBA: Ubiquitin-associated domain
NAF: National Arbitration Forum Domain
KIN10: SNF1 kinase homolog 10 protein kinase
KIN11: SNF1 kinase homolog 11 protein kinase
OST1: OPEN STOMATA 1 protein kinase
CIPK: Calcineurin B like protein-interacting protein kinase
SOS2: SALT OVERLY SENSITIVE 2 protein kinase
bZIP63: BASIC LEUCINE ZIPPER 63 Transcription factor
ABA: Abscisic acid
qRT-PCR: Quantitative real-time polymerase chain reaction
