# Supplementary material for: Identification and characteristics of SnRK genes and cold stress-induced expression profiles in Liriodendron chinense
Source: BMC Genomics. 2022 Oct 18;23:708. doi: 10.1186/s12864-022-08902-0 (PMC9578244; doi:10.1186/s12864-022-08902-0)
Supplement: Supplementary file 2 — Additional file 2: Fig. S1. Phylogenetic tree of LcSnRKs. The scale of the length of each branch is labeled at the bottom of the graph, and the bootstrap value of each branch is labeled at the node position. [file 12864_2022_8902_MOESM2_ESM.docx]

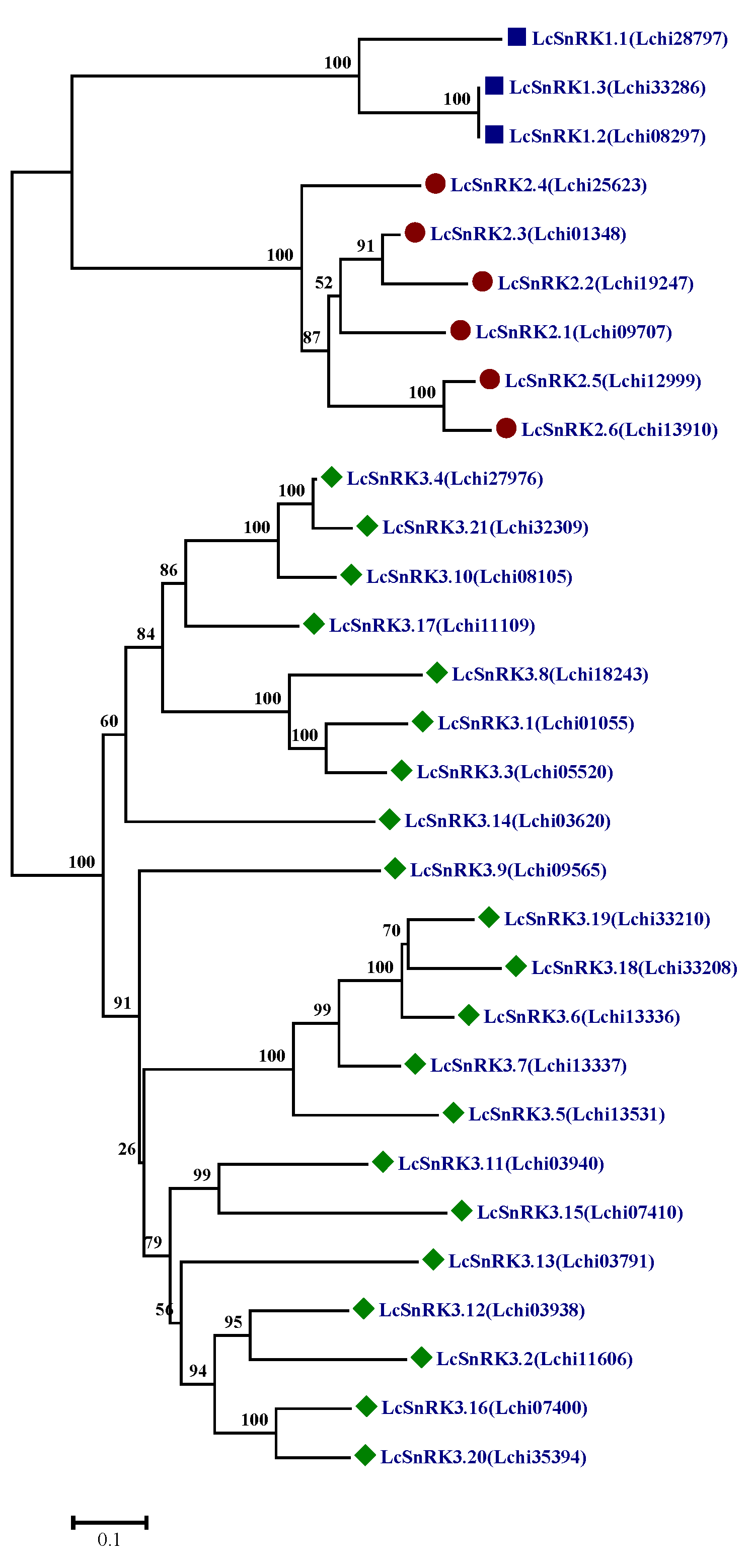


**Figure S1.** *LcSnRK* phylogenetic tree. The scale of the length of each branch is labeled at the bottom of the graph, and the bootstrap value of each branch is labeled at the node position.
