# Supplementary material for: Identification and characteristics of SnRK genes and cold stress-induced expression profiles in Liriodendron chinense
Source: BMC Genomics. 2022 Oct 18;23:708. doi: 10.1186/s12864-022-08902-0 (PMC9578244; doi:10.1186/s12864-022-08902-0)
Supplement: Supplementary file 4 — Additional file 4: Fig. S2. 15 Conservative motifs logo of LcSnRKs. [file 12864_2022_8902_MOESM4_ESM.docx]

| **Motif number** | **Sequences** | **E-value** | **Motif logo** |
| --- | --- | --- | --- |
| Motif 1 | [GE]YDG[AKS]KAD[VIL]WSCGV[IT]L[FY]V[LM][LM][AV]GY[LY]PF[DE]D[SP][ND][LI][PM]X[LMN][YF][RK]K | 2.3e-728 | **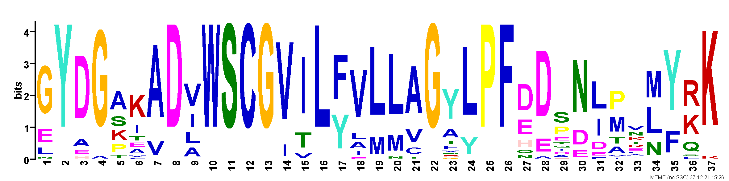** |
| Motif 2 | AR[RK][YF]FQQLIS[AG][VI]D[YF]CHS[RN]GVYHRDLKPEN | 6.9e-577 | **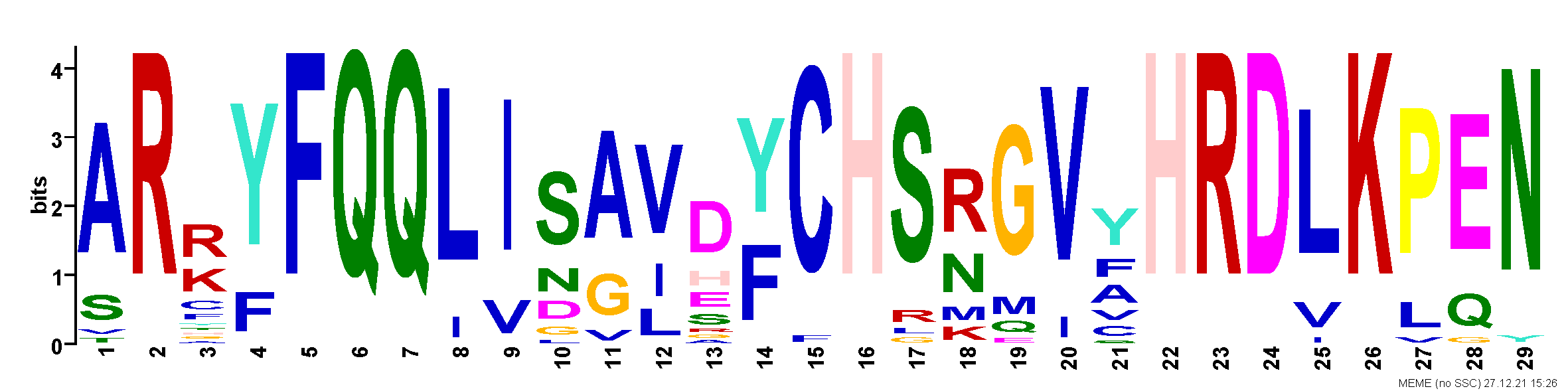** |
| Motif 3 | KRE[IV]S[IV]M[RK][LR][LV]RHPN[IV][VI][REQ]L[YH]EVMA[TS]K[TS]KI[YF][IFL]V[ML] | 3.0e-423 | **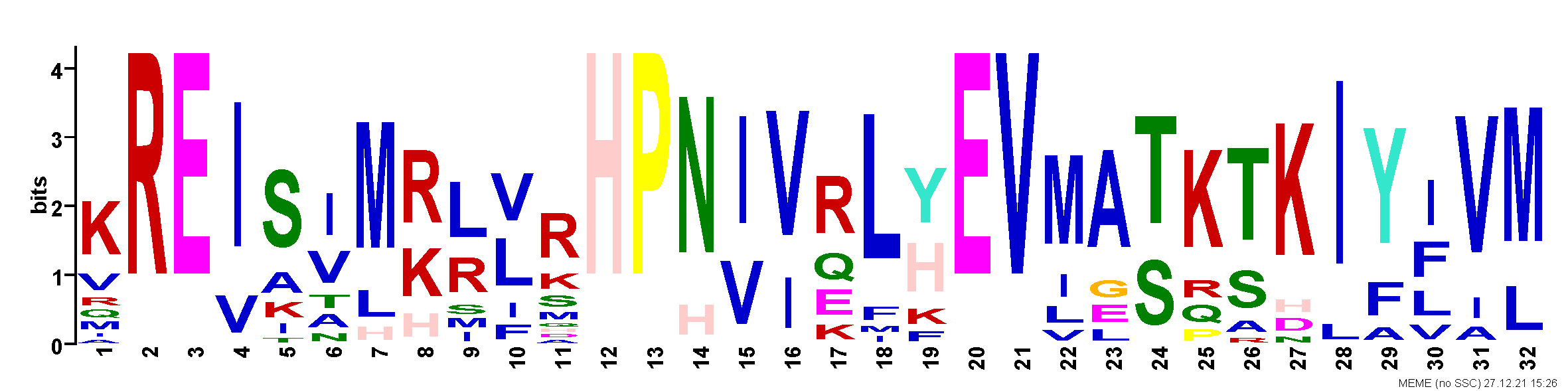** |
| Motif 4 | [DH][GS]L[LP][HK][TS][TA][CV]GTP[AN]Y[VI]APEV[LI][SN][RK]K | 9.8e-383 | **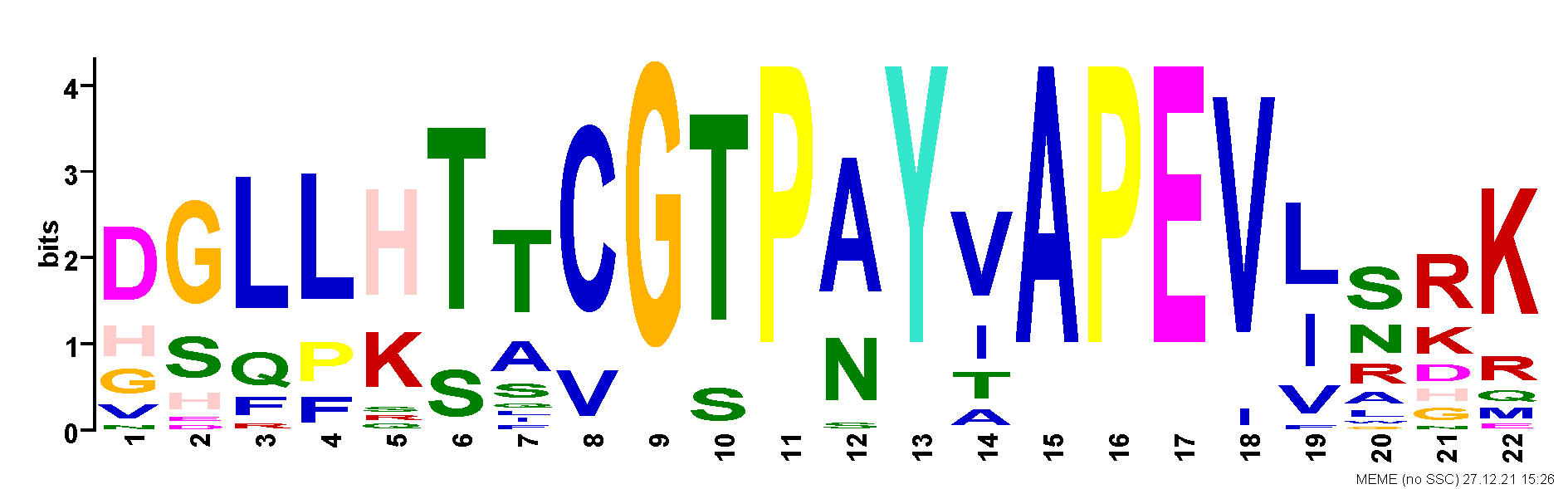** |
| Motif 5 | [FL]SP[GE][AVC]R[RHK]L[IL][ST]R[IL][LF][DV][PA][ND]PS[TK]RITIPE[IL][MR]E[HD][PE]WF[KL]K[GN][LFY]P | 4.0e-462 | **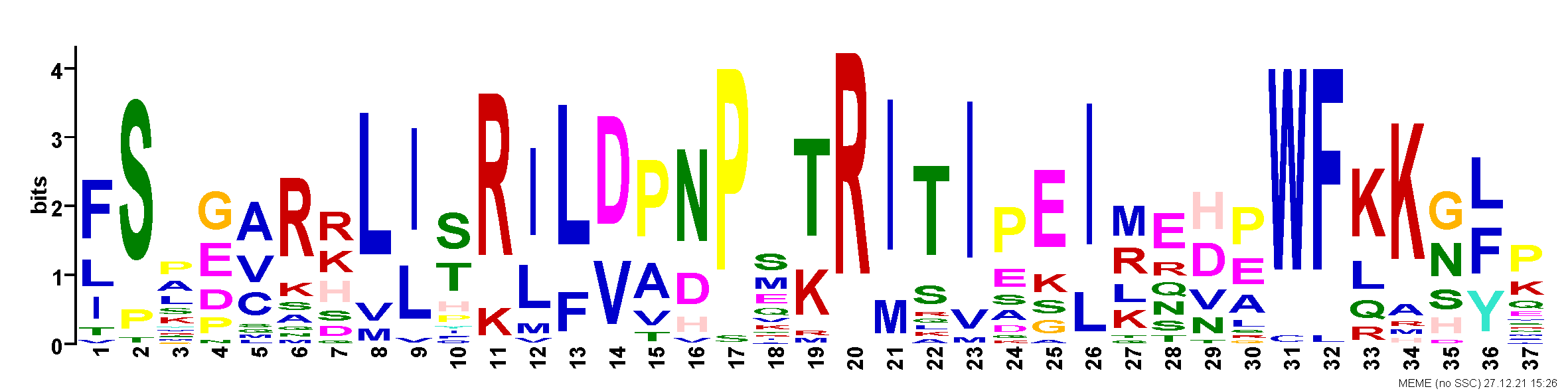** |
| Motif 6 | [TN]F[AG]KV[YK][FHL]ARNLE[TS]GESVA[IV]K[VI][IL]D[KR]EK[IV][LK][KN] | 8.4e-320 | **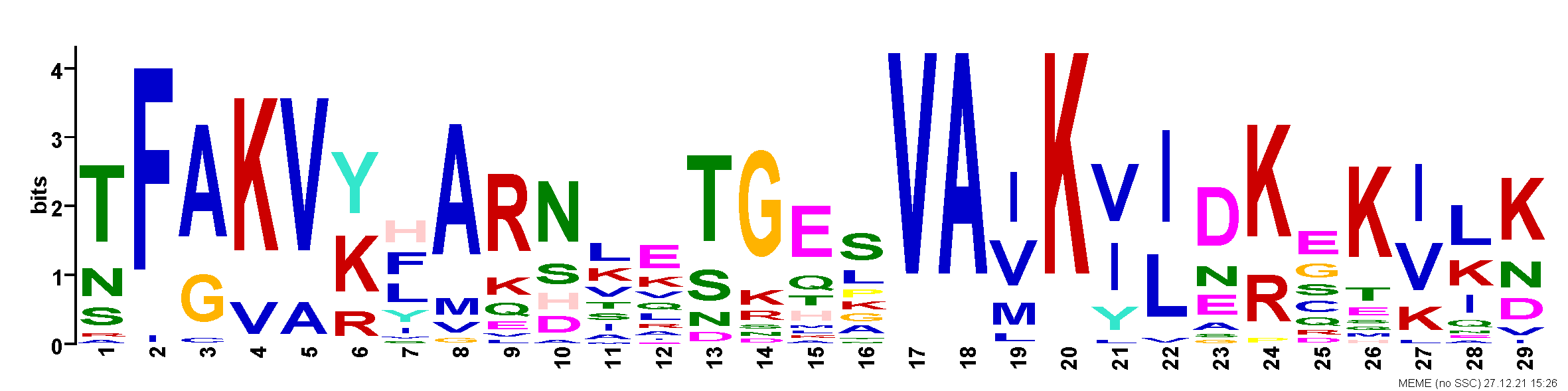** |
| Motif 7 | L[LD][LG][DS][ES]N[GP][NR]LK[VI][SCT]DFG[LY]S[AK][LS][SP][ER] | 1.5e-307 | **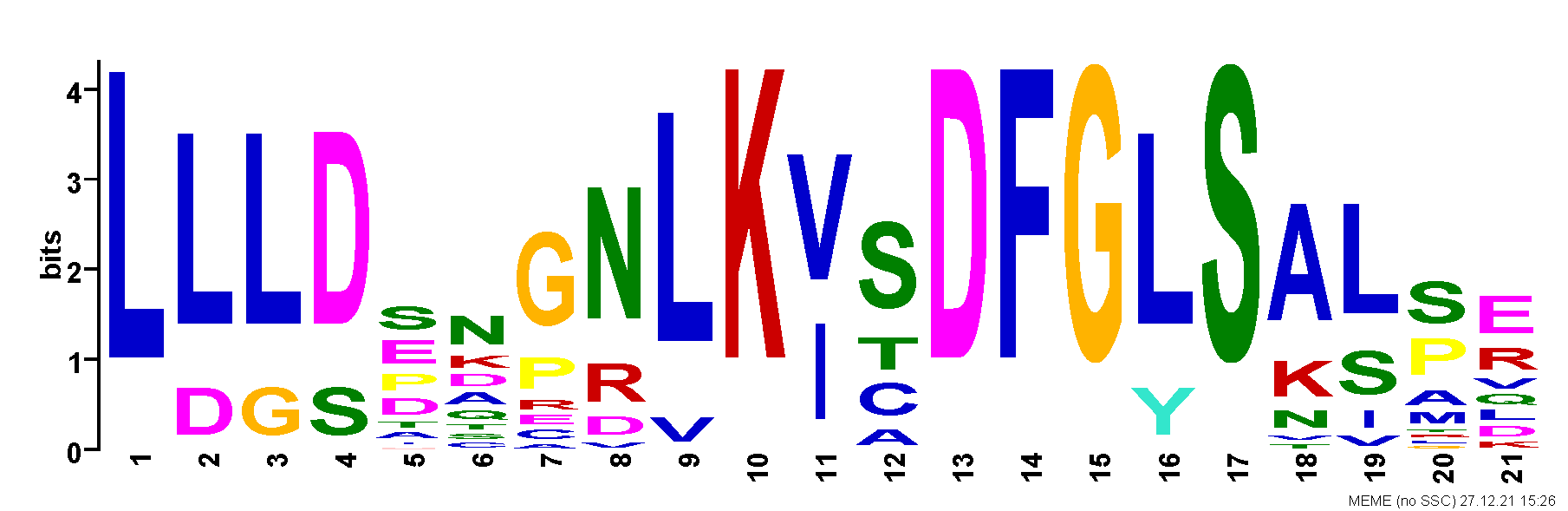** |
| Motif 8 | [KV][EA][GL]RKG[QN]L[ASV][IVL][SD][AVT]E[IV][FS]E[VM][AT]PSL[HFL][ML]VEV[KR]K[SA][AG]GDT[LA]E[FY][HEQ][KE]F[YC][KN] | 1.0e-230 | **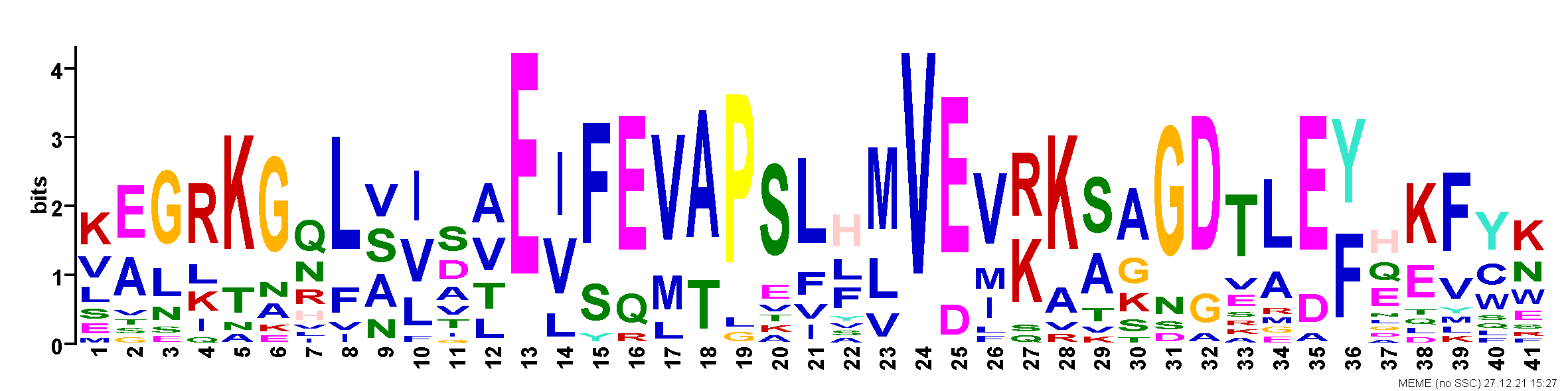** |
| Motif 9 | E[YF][VA][TK]GGELF[DS]K[IV][AV]K[HG] | 1.5e-142 | **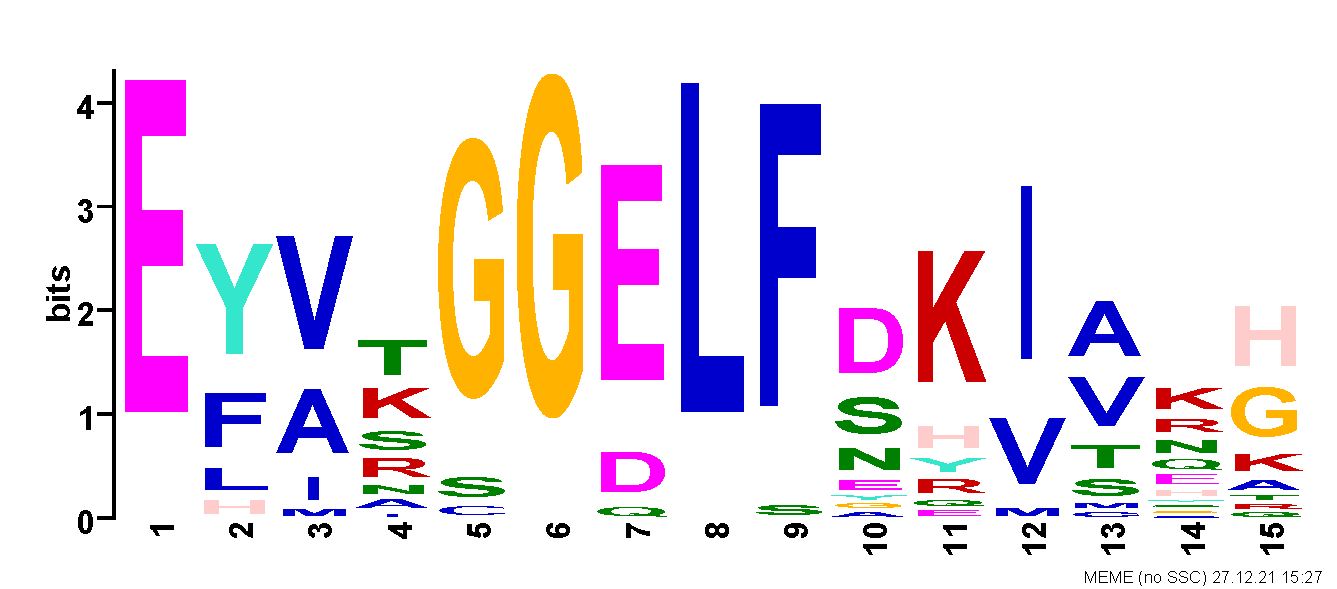** |
| Motif 10 | [IL]IS[LM]S[SQ]G[LF][DN]LSGLFE | 1.5e-127 | **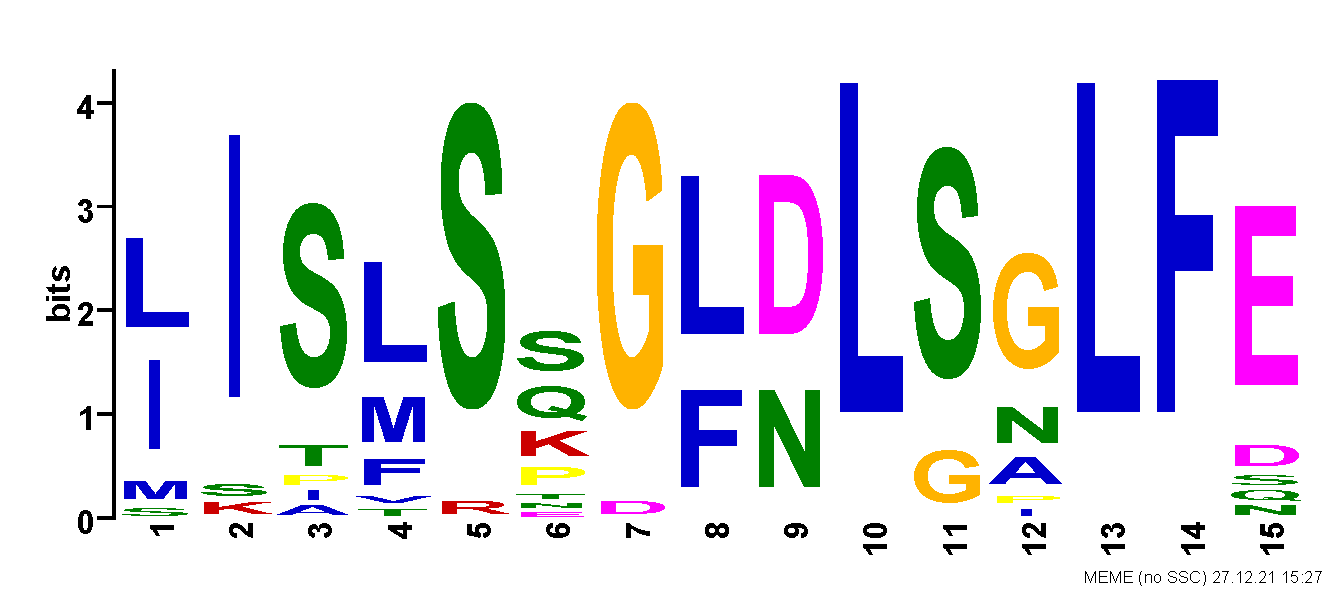** |
| Motif 11 | KRE[TK]RFTS[KTR][KC]PA[KNS][ADE]I[ILV][SE]K[LI]EE | 1.5e-121 | **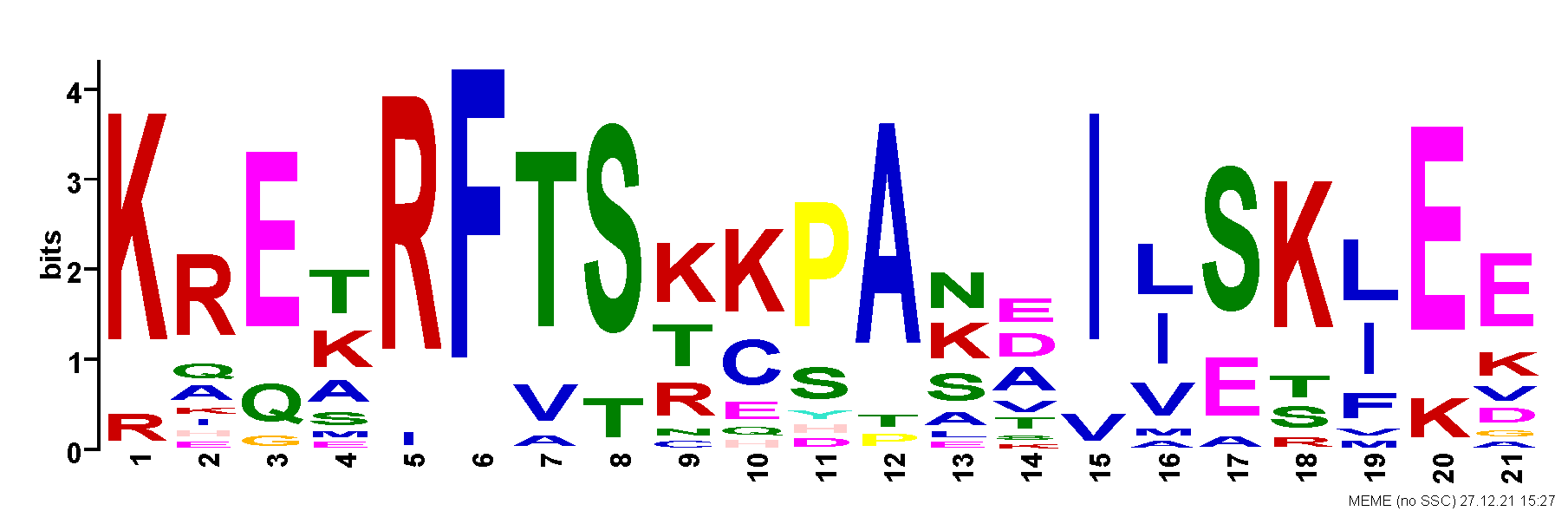** |
| Motif 12 | I[SY][RK][AGV][EDQ][FY][KS][CFI]P[PD][WY] | 7.7e-109 | **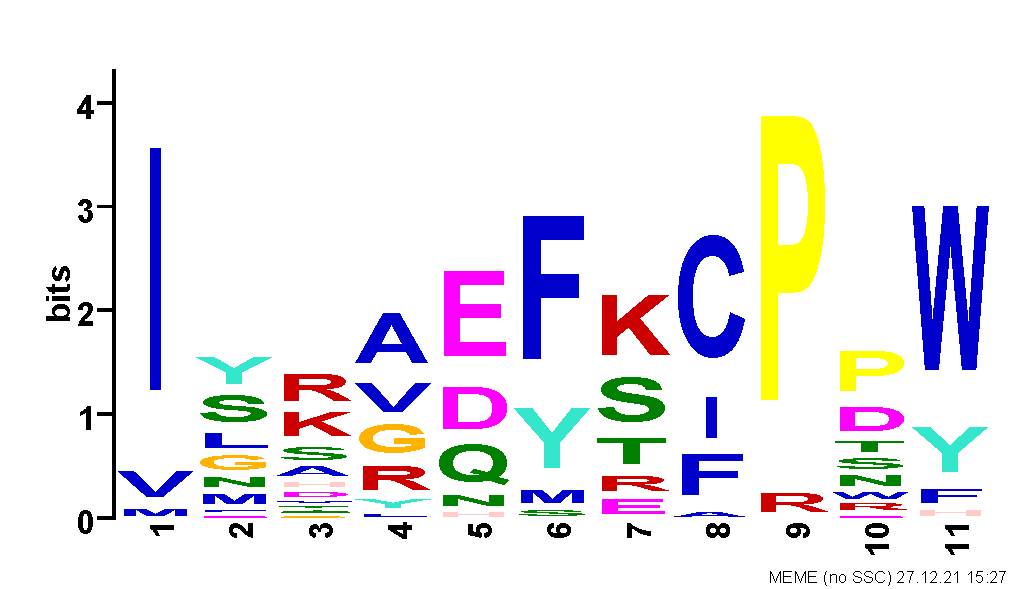** |
| Motif 13 | [KR]YELG[RK][LT][LI]G[ES]G | 9.1e-107 | **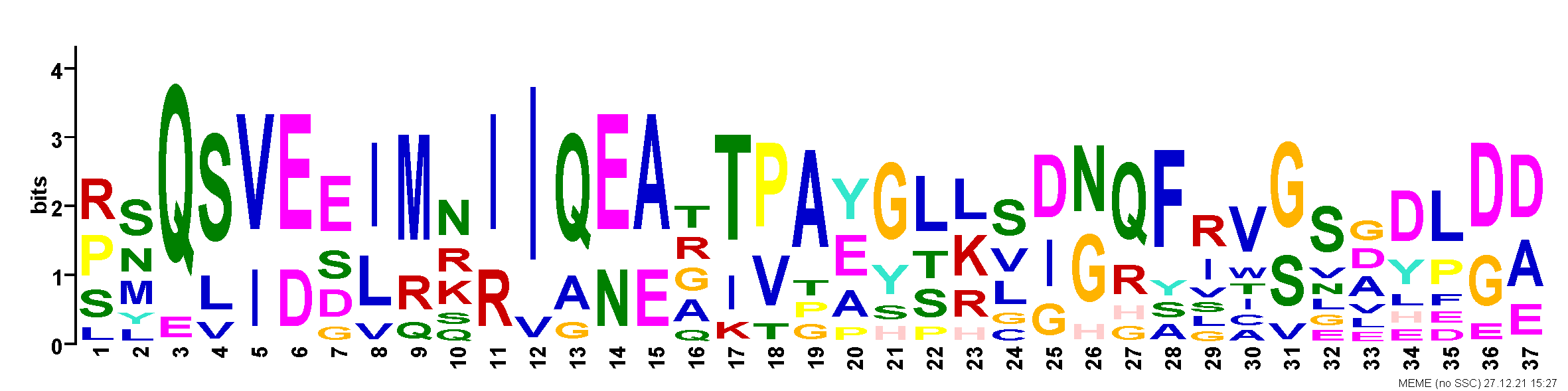** |
| Motif 14 | [PRS][SMN]Q[SL][VI][ED][EDS][IL][MR][NKR][IR]I[QA][EN][AE][AGRT][TI][PV]A[EYA][GY][LST][KLR][SLV][DIG][GN][QR]F[RI]V[GS]S[ADG][DY][LP][DG][DAE] | 1.2e-049 | **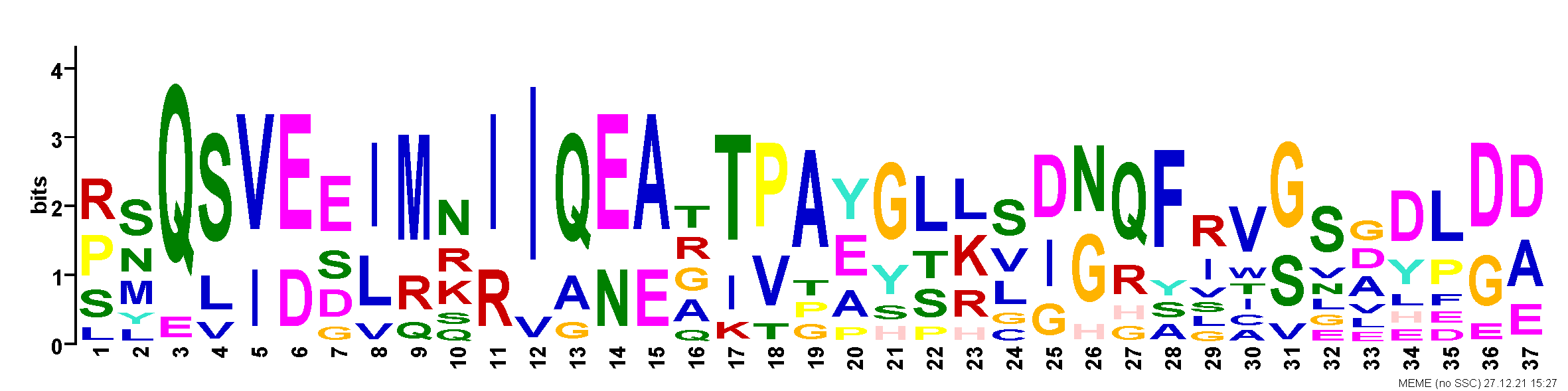** |
| Motif 15 | A[TM][PL]GCMNPDL[PL]LH[ED]NQPNLC[MV]LQNDG[EHR]FN[LH]VN[GR]APVMTAFD | 1.9e-044 | **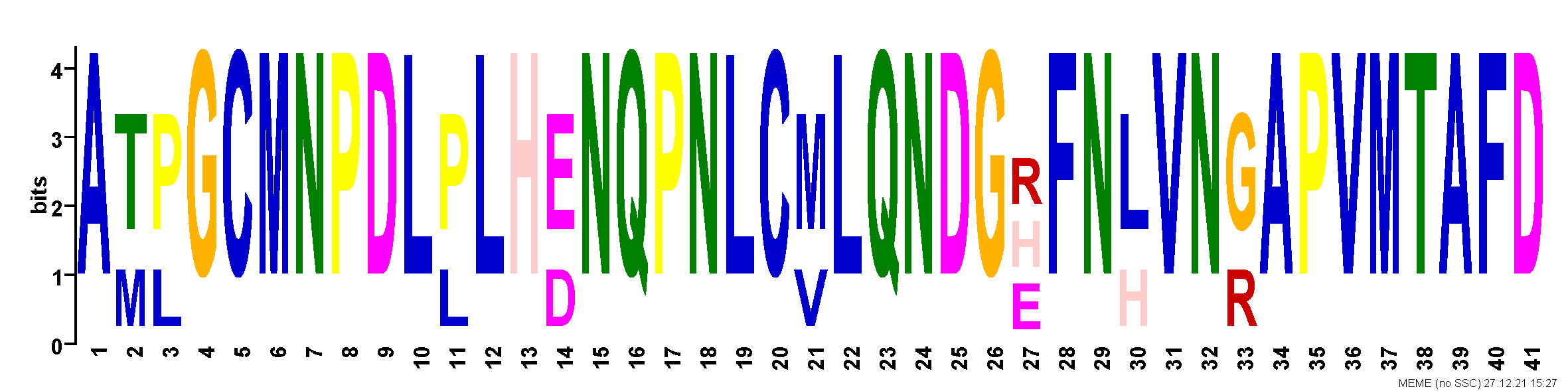** |

**Figure S2.** 15 conservative motifs logo of *LcSnRK*.
