# Supplementary figures and images for: Identification and characteristics of SnRK genes and cold stress-induced expression profiles in Liriodendron chinense
Source: BMC Genomics. 2022 Oct 18;23:708. doi: 10.1186/s12864-022-08902-0 (PMC9578244; doi:10.1186/s12864-022-08902-0)

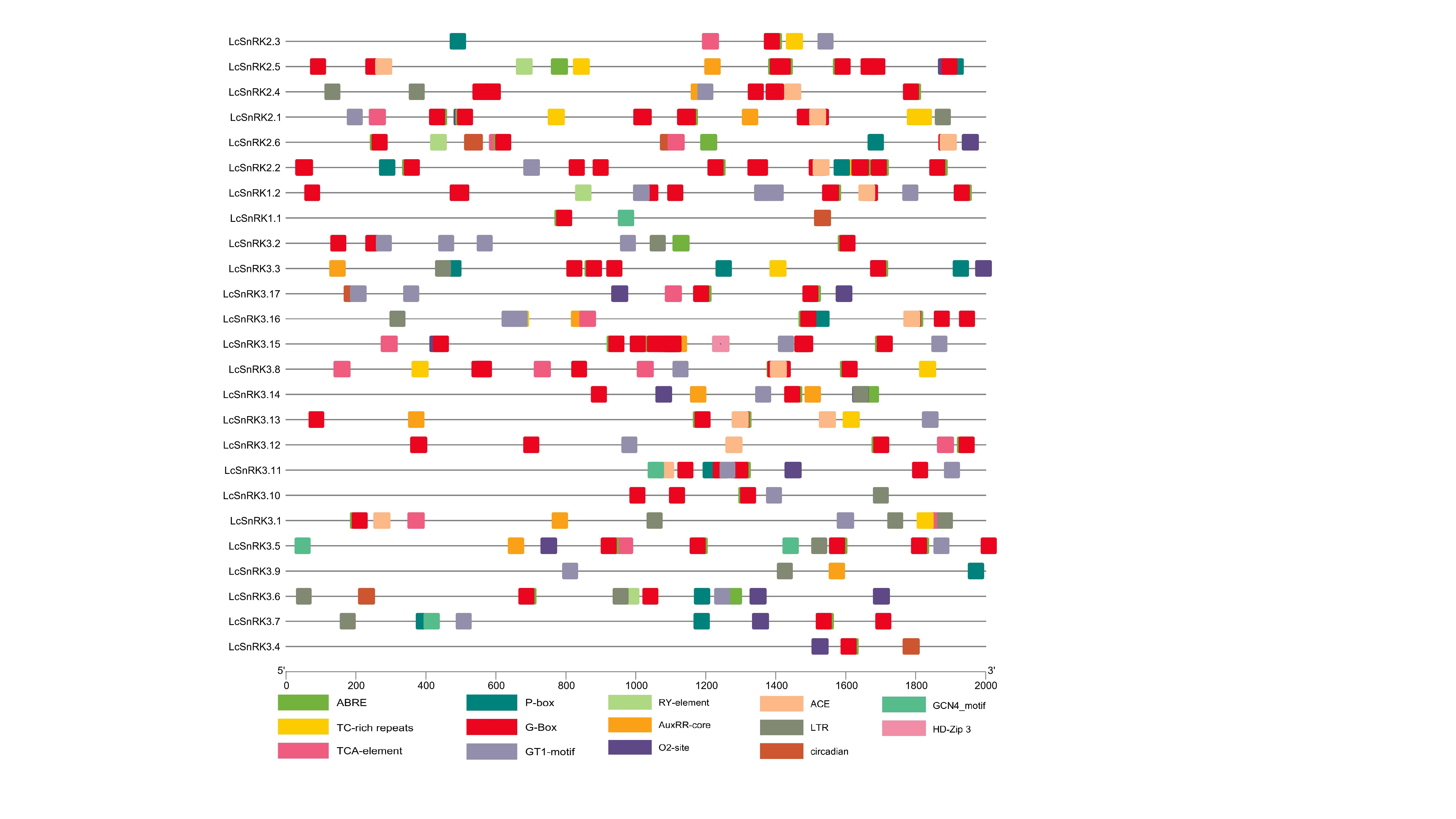


**Figure S3.** The detailed distribution of each cis-acting element in the promoter region.

Supplement: Supplementary file 5 — Additional file 5: Fig. S3. The detailed distribution of each cis-acting element in the promoter region. [file 12864_2022_8902_MOESM5_ESM.docx]
