# Supplementary material for: Identification and characteristics of SnRK genes and cold stress-induced expression profiles in Liriodendron chinense
Source: BMC Genomics. 2022 Oct 18;23:708. doi: 10.1186/s12864-022-08902-0 (PMC9578244; doi:10.1186/s12864-022-08902-0)
Supplement: Supplementary file 12 — Additional file 12: Fig. S5. Sequence alignment of AtOST1 and LcSnRK2.3 protein. Conservative sites in the activation loop are indicated by a red asterisk. Differently colored line segments highlight completely conservative and potential phosphorylated residues. [file 12864_2022_8902_MOESM12_ESM.docx]

**
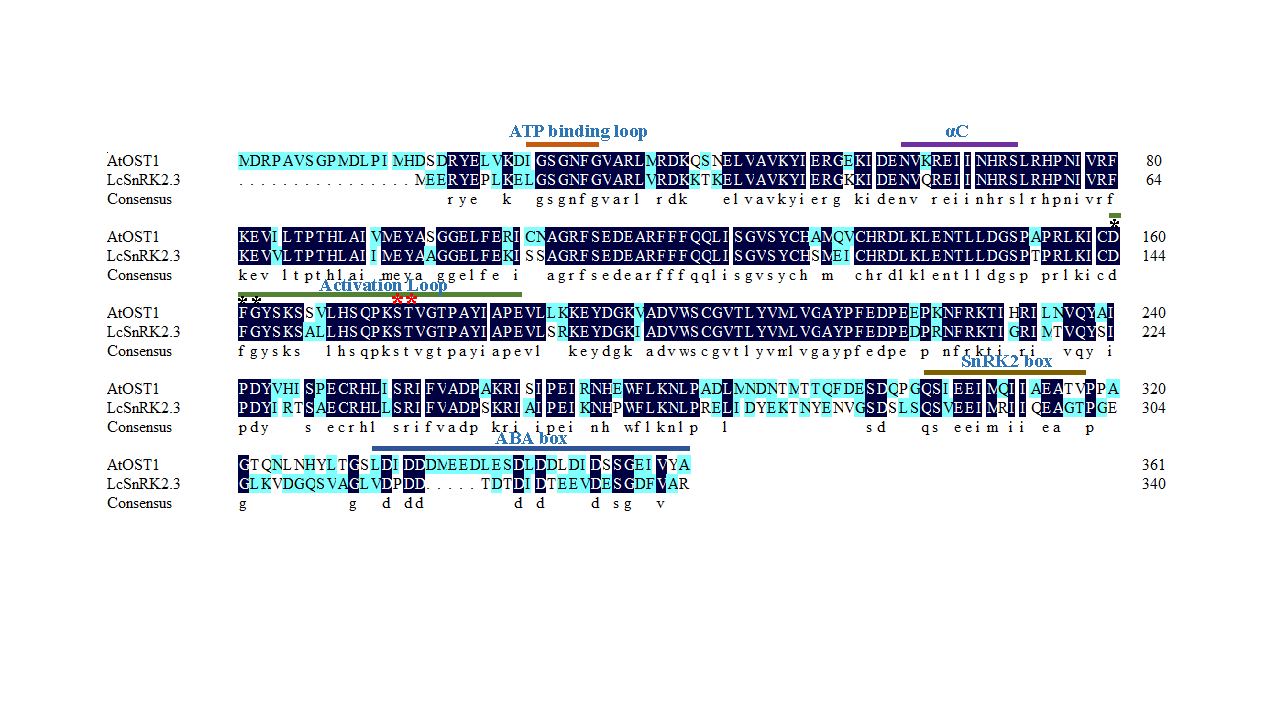
**

**Figure S5.** Sequence alignment of *AtOST1* and *LcSnRK2.3* protein. Conservative sites in the activation loop are indicated by a red asterisk. Differently colored line segments highlight completely conservative and potential phosphorylated residues.
