# Supplementary material for: Identification and characteristics of SnRK genes and cold stress-induced expression profiles in Liriodendron chinense
Source: BMC Genomics. 2022 Oct 18;23:708. doi: 10.1186/s12864-022-08902-0 (PMC9578244; doi:10.1186/s12864-022-08902-0)
Supplement: Supplementary file 13 — Additional file 13: Fig. S6. Sequence alignment of AtSOS2 and LcSnRK3.1 proteins. Differently colored line segments highlight completely conservative and potential phosphorylated residues. [file 12864_2022_8902_MOESM13_ESM.docx]

**
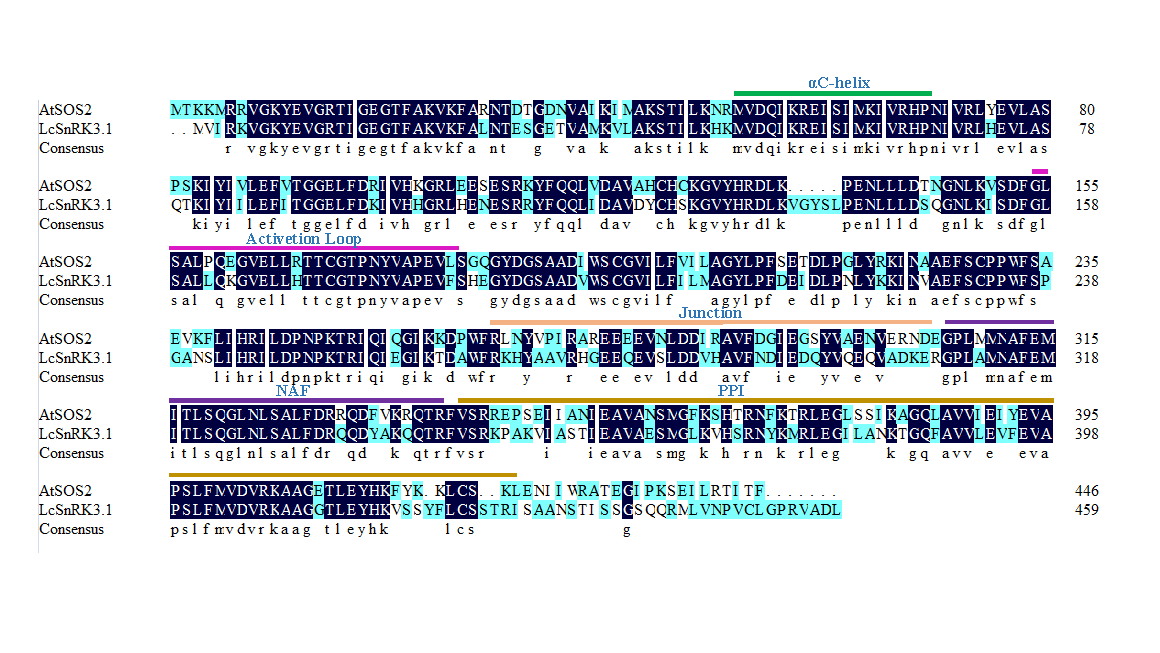
**

**Figure S6.** Sequence alignment of *AtSOS2* and *LcSnRK3.1* proteins. Differently colored line segments highlight completely conservative and potential phosphorylated residues.
